# Responsiveness and interpretability of commonly used outcome assessments of mobility capacity in older hospital patients with cognitive spectrum disorders

**DOI:** 10.1186/s12955-021-01690-3

**Published:** 2021-03-01

**Authors:** Tobias Braun, Christian Thiel, Ralf-Joachim Schulz, Christian Grüneberg

**Affiliations:** 1 Division of Physiotherapy, Department of Applied Health Sciences, Hochschule für Gesundheit (University of Applied Sciences), Gesundheitscampus 6-8, 44801 Bochum, Germany; 2 Training and Exercise Science, Faculty of Sports Science, Ruhr-University Bochum, Bochum, Germany; 3 Department of Geriatric Medicine, St. Marien-Hospital, Kunibertskloster 11-13, 50668 Cologne, Germany

**Keywords:** Older people, Mobility limitation, Dementia, Cognitive impairment, Outcome assessment, Responsiveness, Minimal important change, Interpretability

## Abstract

**Background:**

In older hospital patients with cognitive spectrum disorders (CSD), mobility should be monitored frequently with standardised and psychometrically sound measurement instruments. This study aimed to examine the responsiveness, minimal important change (MIC), floor effects and ceiling effects of commonly used outcome assessments of mobility capacity in older patients with dementia, delirium or other cognitive impairment.

**Methods:**

In a cross-sectional study that included acute older hospital patients with CSD (study period: 02/2015–12/2015), the following mobility assessments were applied: de Morton Mobility Index (DEMMI), Hierarchical Assessment of Balance and Mobility (HABAM), Performance Oriented Mobility Assessment, Short Physical Performance Battery, 4-m gait speed test, 5-times chair rise test, 2-min walk test, Timed Up and Go test, Barthel Index mobility subscale, and Functional Ambulation Categories. These assessments were administered shorty after hospital admission (baseline) and repeated prior to discharge (follow-up). Global rating of mobility change scales and a clinical anchor of functional ambulation were used as external criteria to determine the area under the curve (AUC). Construct- and anchor-based approaches determined responsiveness. MIC values for each instrument were established from different anchor- and distribution-based approaches.

**Results:**

Of the 63 participants (age range: 69–94 years) completing follow-up assessments with mild (Mini Mental State Examination: 19–24 points; 67%) and moderate (10–18 points; 33%) cognitive impairment, 25% were diagnosed with dementia alone, 13% with delirium alone, 11% with delirium superimposed on dementia and 51% with another cognitive impairment. The follow-up assessment was performed 10.8 ± 2.5 (range: 7–17) days on average after the baseline assessment. The DEMMI was the most responsive mobility assessment (all AUC > 0.7). For the other instruments, the data provided conflicting evidence of responsiveness, or evidence of no responsiveness. MIC values for each instrument varied depending on the method used for calculation. The DEMMI and HABAM were the only instruments without floor or ceiling effects.

**Conclusions:**

Most outcome assessments of mobility capacity seem insufficiently responsive to change in older hospital patients with CSD. The significant floor effects of most instruments further limit the monitoring of mobility alterations over time in this population. The DEMMI was the only instrument that was able to distinguish clinically important changes from measurement error.

***Trial registration*:**

German Clinical Trials Register (DRKS00005591). Registered February 2, 2015.

## Background

Cognitive spectrum disorders (CSD) is a term that encompasses diagnosed dementia, delirium, delirium superimposed on known dementia and other unspecified cognitive impairments [1]. Patients with CSD constitute a significant proportion of older hospital patients, and the number of people with dementia is expected to rise significantly within the next decades [2]. Today, the in-hospital prevalence of dementia is estimated to be between 13 and 63% [3], and as many as 50% of people older than 65 years of age who are admitted to hospitals present with delirium [4]. Reynish et al. [1] reported a 39% prevalence of CSD in older adults admitted to an emergency department.

Mobility is defined as ‘moving by changing body position or location or by transferring from one place to another, by carrying, moving or manipulating objects, by walking, running or climbing, and by using various forms of transportation’ [5]. Mobility capacity is a relevant indicator of the health status and the quality of life of older people [6]. In older hospital patients, however, mobility impairments are common and associated with a risk of additional loss of function [7]. Approximately 30–60% of older medical patients are not able to stand or walk without physical assistance at hospital admission [8–10]. Mobility decline is also considered an undesirable disease presentation that may facilitate risk stratification in older people admitted to hospitals [11].

The goal of mobility assessment is to guide interventions supporting mobility and, thus, to improve care [12]. Mobility should be assessed frequently and with standardised and psychometrically sound measurement instruments [11, 12], in terms of reliability, validity and responsiveness to change [13, 14]. To assign qualitative meaning to a measurement instrument’s quantitative scores or change in scores, aspects of interpretability such as minimal important change (MIC) values or floor and ceiling effects in a specific population are of special interest [14].

Reviews and recommendation statements have outlined many multi-component mobility capacity measures that are considered suitable for older hospital patients [12, 15–17], including the Hierarchical Assessment of Balance and Mobility (HABAM) [18], the Short Physical Performance Battery (SPPB) [19], Tinetti’s Performance Oriented Mobility Assessment (POMA) [20] and the de Morton Mobility Index (DEMMI) [21]. In clinical practice, (shorter) single-component measures of mobility are also used frequently [22, 23], such as timed short- and long-distance gait measures, timed chair rise tests and the Timed Up and Go test (TUG) [24]. However, there is no ‘gold standard’ or widely accepted consensus on a specific measurement instrument of mobility capacity for acute older medical patients in inpatient settings [12].

In clinical care and research, mobility measures are often used to monitor a patient’s individual progress or disease progression and to evaluate the effect of interventions, such as exercises [25]. For these objectives, a measurement instrument must be sufficiently responsive. Responsiveness to change, which is defined as ‘the ability of an outcome measure to detect change over time in the construct to be measured’ [14], is the measurement property that has been examined the least in older (hospitalized) individuals [15, 17], and especially in those with cognitive impairment [26–28]. Because of a lack of psychometric studies, McGough et al. [26] calculated effect sizes, as an indicator of responsiveness, from data reported in clinical trials on exercise interventions in older people with dementia. The authors [26] found that the 6-min walk test, the TUG, repeated chair stand tests and short-distance gait speed tests were the most frequently used outcome measures of mobility capacity. These measurement instruments demonstrated a small, medium or large effect in at least 50% of exercise intervention studies [26]. However, these results provide only limited evidence of responsiveness, since the assessment of responsiveness on the basis of effect size is considered invalid [13, 29, 30]. Effect size indices were developed as standardised measures of the magnitude of the effect of an intervention or another event that happened over time; therefore, expressing the magnitude of change relative to the standard deviation (SD) [13]. Thus, ‘a high magnitude of change gives little indication of the ability of the instrument to detect change over time on the construct to be measured’ [13]. In the absence of high-quality psychometric studies and systematic reviews, the responsiveness of commonly used measurement instruments of mobility capacity in older hospital patients with CSD is largely unknown.

For planning and evaluating healthcare interventions, valid information on the interpretability of a patient’s mobility test scores is crucial. The MIC, which is defined as ‘the smallest change in score in the construct to be measured which patients perceive as important’ [13, 14], is a key parameter of interpretability in clinical care. Knowledge of the MIC of a measurement instrument helps to interpret the relevance of measured changes. It also provides a metric for the planning of sample sizes in clinical trials based on the proportion of patients reaching the MIC or higher [31]. The MIC values of measurement instruments of mobility capacity in older hospital patients with CSD are largely unknown [26, 27].

In older hospital patients with CSD, the valid monitoring of mobility alterations is especially challenging; for example, complex test instructions and a high prevalence of functional limitations in this population [26, 32, 33] lead to significant floor effects of single-component measures, such as timed walk tests [8, 11, 34]. Although floor and ceiling effects can significantly affect the clinical value of mobility measures in older hospital patients with CSD, there is very limited evidence on these aspects of interpretability.

We have recently examined the psychometric properties of the DEMMI in older individuals with dementia, delirium, or other cognitive impairments, providing the first evidence that the DEMMI is a feasible, unidimensional and construct-valid measurement instrument of mobility in this population [35]. The DEMMI was also found to be free of floor and ceiling effects [35]. In a sub-analysis of the primary study, we have further analysed the test–retest reliability of the DEMMI and other commonly used mobility measures in older people with CSD [36]. The results indicated sufficient test–retest reliability for group-comparisons in all examined instruments, but limited use for individual monitoring of mobility over time due to the large measurement error in most of the instruments.

Since responsiveness and MIC of the DEMMI have not yet been analysed in older individuals with CSD, the main objective of the present study was to assess these measurement properties. Given the lack of evidence on responsiveness, MIC values, and floor and ceiling effects of mobility measures in older hospital patients with CSD, the secondary objective of the present study was to determine these measurement properties for several other commonly used measures of mobility capacity in this population based on the available data set.

## Methods

### Design and setting

Some methodical aspects of this study have already been reported elsewhere [35, 36]. The primary study [35] was approved by the Ethical Review Board of the University of Cologne (registration number 2014-05), conducted according to the ethical principles of the Declaration of Helsinki (2013), a priori registered in the German Clinical Trials Register (DRKS00005591) and performed in a geriatric hospital in Cologne, Germany (St. Marien-Hospital) [35, 36]. All participants provided written and ongoing informed consent, according to previously reported procedures. Recommendations of the STrengthening the Reporting of Observational studies in Epidemiology (STROBE) statement for cross-sectional studies were followed. Reporting was further informed by the criteria of the Consensus-based Standards for the selection of health Measurement Instruments (COSMIN) risk-of-bias checklist [37].

Participants with CSD included in the primary study (n = 153) [35] were assessed with a comprehensive set of mobility measures immediately after hospital admission (baseline sample). A sub-sample of the baseline participants repeated all baseline mobility measures [23, 35]. The present study reports the responsiveness and MIC values of commonly used measurement instruments of mobility capacity and physical functioning.

### Participants

Participant were enrolled from February 4, 2015 to December 11, 2015 [35, 36]. During the study period, we defined 91 screening days, which were spread unsystematically [35]. All acute older inpatients consecutively admitted to the hospital on one of the screening days were screened for eligibility. A sample of 153 patients was included and constituted the baseline sample of the primary study [35, 36].

Patients were eligible if they were admitted to one of the acute geriatric wards of the hospital, ≥ 60 years old, and presented with a cognitive impairment, as indicated by a Mini-Mental State Examination (MMSE) score of ≤ 24 points [38]. The exclusion criteria included: documented contraindications for mobilisation, physician-directed partial weight-bearing of the lower extremity, isolation for infection, impending death, coma or severely impaired vigilance, acute major organ failure, blindness, deafness, severe dysphasia, a German-language barrier, or any acute psychiatric or medical/physical condition whereby mobility measurements could lead to a worsening of the patient’s state of health [35, 36].

For the follow-up assessment, participants were excluded if they (1) were discharged within 6 days after the baseline assessment, (2) refused a second assessment (3) or were in an unstable/critical medical condition.

### Procedures

Eligible participants were examined within 7 days after hospital admission (baseline assessment). In a single baseline session, a comprehensive set of commonly used performance-based measurement instruments of mobility capacity was administered in a standardised order, starting with the least physically challenging tests. The procedure has been reported in detail previously [35, 36].

Participants were invited to participate in a follow-up session including the same set of measurement instruments used in the baseline assessment. The measurements were performed by the same rater, in the same order, and under the same conditions as in the baseline assessment.

The follow-up assessment was scheduled as close as possible to the patient’s hospital discharge and took place 7–21 days after the baseline assessment. A minimum of 7 days was chosen, since we expected a significant proportion of patients to experience changes in their mobility capacity over this period and still reassess a maximum number of participants before discharge [13]. Socio-demographic data was taken from the medical records and from hospital administrative data [35, 36].

### Measurements

In this study [35, 36], 10 performance-based measures of the mobility capacity of older people were applied in the following order: DEMMI [21, 34], HABAM [39, 40], POMA [20], TUG [24], SPPB [19], 4-m gait speed test (as part of the SPPB), 5-times chair rise test (5xCRT; as part of the SPPB), 2-min walk test [41], Barthel Index mobility subscale [42], and Functional Ambulation Categories (FAC) [43].

We clustered all measurement instruments examined in this study according to the ICF mobility domain components captured by each instrument [36]. Accordingly, instruments are separated into single- and multi-component measures depending on the number of mobility domains included. Table 1 presents a clustered overview, including each instrument’s scale range [36]. The classification is the consensus of the authors, informed by the classifications reported by other authors [17, 44]. Additional file 1 provides a detailed description of the assessment procedures and all measurement instruments.

**Table 1:** Mobility domain components of each measurement instrument classified according to the ICF

Domain components		Multi-component measurement instruments						Single-component measurement instruments			
		DEMMI	HABAM	POMA	SPPB	TUG	Barthel Index mobility subscale	FAC	4-m gait speed test	5x chair rise test	2-min walk test
		0–100 points	0–26 points	0–28 points	0–12 points	Ratio scale (s)	0–40 points	0–5 points	Ratio scale (m/s)	Ratio scale (s)	Ratio scale (m)
Changing and maintaining body position (d410–d429)											
d410	Changing basic body position	X	X	X	X	X	X			X	
d415	Maintaining a body position	X	X	X	X						
d420	Transferring oneself		X				X				
Carrying, moving and handling objects (d430–d449)											
d430	Lifting and carrying objects										
d435	Moving objects with lower extremities										
d440	Fine hand use										
d445	Hand and arm use										
Walking and moving (d450–d469)											
d450	Walking	X	X	X	X	X	X	X	X		X
d455	Moving around		X				X				
d460	Moving around in different locations										
d465	Moving around using equipment	X	X	X		X	X				
Moving around using transportation (d470–d489)											
d470	Using transportation										
d475	Driving										
d480	Riding animals for transportation										

The domain components (constructs) of each instrument are classified according to the domain ‘mobility’ (Activities and Participation, Chapter 4) described in the World Health Organization’s ICF [5]. The ICF mobility definition is: ‘Moving by changing body position or location or by transferring from one place to another, by carrying, moving or manipulating objects, by walking, running or climbing, and by using various forms of transportation’

*ICF* International Classification of Functioning, Disability and Health, *DEMMI* de Morton Mobility Index, *HABAM* Hierarchical Assessment of Balance and Mobility, *POMA* Performance Oriented Mobility Assessment, *SPPB* Short Physical Performance Battery, *TUG* Timed Up and Go test, *FAC* Functional Ambulation Categories

#### Patient-reported global rating of change amount (P-GRC-A) scale

After the follow-up assessment, a short ICF definition of mobility was provided to the participants. Then, participants were asked if their mobility had improved, deteriorated or remained unchanged since the baseline assessment (hospital admission). If participants reported improvement, they were asked to estimate the *amount of mobility change* (improvement or deterioration) on a 5-point global rating of change (P-GRC-A) scale ranging from ‘a little bit’, ‘somewhat’, ‘moderately’, ‘much’ to ‘very much’ *better* (+ 1 to + 5). Participants who reported deterioration were given a corresponding scale (e.g. ‘a little bit’ to ‘very much’ *worse*; − 1 to − 5).

We used independent scales for participant improvement and deterioration due to their better feasibility with older participants. This approach is indeed consistent with an 11-point global rating of change scale (− 5 to + 5).

#### Patient-reported global rating of change importance (P-GRC-I) scale

Participants who reported any change in mobility were asked to estimate the *importance of mobility change* (improvement or deterioration) on a 6-point global rating of change scale (P-GRC-I), ranging from ‘unimportant’, ‘a little’, ‘somewhat’, ‘moderately’, ‘quite’ to ‘very’ *important* (0 to + 5). For example, a participant who estimated the *amount* of mobility change to be ‘moderate’ (P-GRC-A = + 3) could rate this change as only ‘a little *important*’ (P-GRC-I = + 1).

#### Therapist-reported global rating of change amount (T-GRC-A) scale

To assess a participant’s mobility change from a clinician’s point of view, assuming more objective estimations, the global rating of change scale procedure described above was performed by each participants’ responsible physiotherapist. In more detail, the physiotherapist was asked if he or she had examined or treated the patient on the days of the baseline and follow-up assessments. If this was not the case, the responsible occupational therapist was consulted. If neither the physiotherapist nor the occupational therapist had seen the participant on both days of the two study measures, the global rating of change scale was not assessable.

Therapists were asked if the mobility of the participant had improved, deteriorated or remained unchanged since the baseline assessment. The *amount* of improvement or deterioration was rated on an 11-point therapist-reported global rating of change (T-GRC-A) scale ranging from − 5 to + 5.

#### Therapist-reported global rating of change importance (T-GRC-I) scale

The same procedure as that for the P-GRC-I scale was followed by asking the therapist to estimate the *importance* of mobility change.

### Statistical analysis

Data were analysed using SPSS 21.0 (IBM Corp.; Armonk, New York, USA) and Microsoft Excel 2016 (Microsoft Office; Redmond, Washington, USA). The sample characteristics are presented descriptively. Interval-based data were examined for normality with the Shapiro–Wilk test of normality and by visual inspection of the related histograms and P–P-plots. *P* < 0.05 indicated statistical significance.

Differences in clinical outcomes at baseline between participants included in this study and participants lost to follow-up were assessed using chi-square tests, t-tests, McNemar tests or Mann–Whitney U tests when appropriate.

The change scores (∆) of all mobility-related measurement instruments were calculated by subtracting the baseline scores from the follow-up scores. Participants who deteriorated according to the anchors were excluded from all analyses on responsiveness and MIC due to the small sample size.

Cohen’s effect size was calculated as the difference between two means divided by the pooled SD.

### Measurement properties

#### Responsiveness

The responsiveness of the 10 mobility measures was assessed following a construct- and an anchor-based approach [14]. The sample size approximation of 150 participants for the baseline sample was based on sample size requirements for a Rasch analysis [35, 45]. For the follow-up measures, we tried to include as many participants as possible, but targeted at least 100 participants [46].

#### Responsiveness: construct approach

Responsiveness was assessed by following the methodological approach of hypotheses testing. Instrument change scores and P-GRC-A and T-GRC-A scores were used to a priori formulate hypotheses [13]. For each instrument listed in Table 1, 11 hypotheses were formulated (H1–H11):

H1–H9: For each instrument, a moderate correlation of ≥ 0.50 between the change scores of this instrument and the change scores of the other nine mobility instruments was expected. The strengths of the correlations were expected to be at least moderate (≥ 0.50), since change scores are accompanied by a high measurement error [13].

H10–H11: For each instrument, a correlation of ≥ 0.30 between the change scores of this instrument and the P-GRA-A and T-GRC-A scores was expected. The strengths of the correlations were expected to be at least weak (≥ 0.30), since global rating of change scales have critical validity and reliability [13, 47] and are known to be subject to recall bias [48]. Furthermore, global rating of change scales are known to be subjected to a high measurement error.

We applied one-tailed Pearson’s r (normally distributed change scores of interval measures) and Spearman’s rho (all other data) analyses, because the directions of the correlations were hypothesized a priori. For instruments in which lower scores represent better functioning (TUG and 5xCRT), a negative correlation was hypothesized. All correlations were reported unidirectionally to improve readability.

We decided against defining an a priori hypotheses percentage threshold (e.g. 75%), which would require confirmation in order for a measurement instrument to be considered valid or responsive [49, 50]. As stated by the COSMIN authors themselves, ‘there is no criterion to decide whether an instrument is valid or responsive. Assessing validity or responsiveness is a continuous process of accumulating evidence’ [30]. That is why we leave it to the reader to decide the percentage of confirmed hypotheses deemed acceptable.

#### Responsiveness: anchor-based approach

We used multiple independent patient-reported and clinical anchors to examine and confirm responsiveness [51]. A correlation threshold of ≥ 0.30 was set as an acceptable association between an anchor and an instrument’s change score [51].

The area under the receiver operating characteristic curve (AUC) for each external anchor was calculated. The AUC can be interpreted as the probability of correctly identifying an improved patient from randomly selected pairs of improved and unchanged patients [52]. An AUC ≥ 70% was considered satisfactory [13, 50].

##### Patient-reported anchor: P-GRC-A scale

The P-GRC-A scale was used as an external anchor for the responsiveness analysis. Participants who rated themselves as a ‘little bit better’ (+ 1), ‘not changed’ (0), or ‘a little bit worse’ (− 1) were labelled ‘unchanged’. Participants who indicated that they were at least ‘somewhat better’ (+ 2 or higher) were labelled ‘improved’.

##### Therapist-reported anchor: T-GRC-A scale

Participants whose amount of mobility change was rated by the therapist to be between − 1 and + 1 on the T-GRC-A scale were deemed ‘unchanged’. Participants with a score of + 2 or higher were deemed ‘improved’.

##### Clinical anchor: functional ambulation categories

The FAC is a rough scale that allows the level of ambulation to be rated according to six categories [43]. We considered a change from one FAC category to the next as a relevant change in mobility. Thus, the FAC anchor was defined as participants who improved their level of ambulation (FAC∆ ≥ 1 points; ‘improved’) versus patients who did not change according to the FAC (FAC∆ = 0 points; ‘unchanged’).

#### Minimal important change (MIC)

There is no consensus on the best method to determine MIC. Generally, a combination of anchor- and distribution-based approaches are recommended and used to reveal a range of values for the MIC [51, 53–56]. Thus, our aim was examining ‘multiple values from different approaches and hopefully converging on a small range of values (or one single value)’ [51]. However, as distribution-based indices provide no direct information on the MIC, these values were only used as supportive information for MIC estimates from anchor-based approaches [51].

#### MIC: anchor-based approach

The MIC was quantified by constructing receiver operating characteristic (ROC) curves [57]. The ROC curve is the result of using different cut-off points for change scores, each with a given sensitivity (*sens*) and specificity (*spec*). The optimal cut-off point (*q*_*f*_) can be used as the MIC value [55, 57, 58]. To estimate MIC thresholds by using cut-off points from ROC curves, different approaches have been proposed. Since no consensus exists, three MIC values (cut-off points) were calculated for each anchor:

1. The method described by Farrar et al. (2001) [59] used the point closest to the intersection of a − 45° tangent line: *q*_*f*_ = min{|*sens* − *spec*|}.
2. Authors from the COSMIN group [57] have proposed to choose the point closest to the top-left corner of the ROC curve, which is assumed to represent the lowest overall misclassification and which is equal to the Youden index [60]: *q*_*f*_ = min{2 − *sens* − *spec*}.
3. Froud et al. (2014) [58] proposed to first square the terms used by COSMIN, giving the following formula: *q*_*f*_ = min{(1 − *sens*)^2^ + (1 − *spec*)^2^}.

Sensitivity and specificity were valued equally. A correlation threshold of a ‘nontrivial’ association (≥ 0.30) [51] was set as an acceptable association between an anchor and an instrument’s change score [51]. Since there is no consensus on a correlation threshold [55, 56, 58] (e.g. the COSMIN authors proposed a ‘substantial’ association without proposing a clear cut-off value [57]), and for the sake of completeness, we also reported MIC values if the rho correlation was < 0.3. However, we highlighted MIC values considered to be invalid according to recent beliefs [51].

A change deemed ‘a little better/worse’ (amount) is not explicitly important in any sense. That is why we used global rating of change scales of *importance* for the MIC analysis. The following external anchors were used to divide the sample into groups of participants who had experienced at least a minimal important change/improvement and participants who experienced an unimportant change/improvement or no change in mobility, according to the anchors.

##### Patient-reported anchor: P-GRC-I scale

Participants who reported no change at all (P-GRC-A = 0) or a change in their mobility of no importance (P-GRC-I = 0) were labelled as ‘not importantly improved’. Participants who rated any perceived improvement (P-GRC-A ≥ + 1) to be at least ‘a little important’ (P-GRC-I ≥ + 1) were labelled as ‘importantly improved’.

##### Therapist-reported anchor: T-GRC-I scale

For the T-GRC-I anchor, the same criteria as for the P-GRC-I anchor were used.

##### Clinical anchor: functional ambulation categories

To calculate the MIC according to the FAC, the same anchor as for the responsiveness analysis was used. Thus, participants with FAC∆ = 0 were considered ‘not importantly improved’, while participants with FAC∆ ≥ 1 were deemed ‘importantly improved’.

#### MIC: within-patient change score approach

Another anchor-based MIC value was determined as the mean change in the instrument change scores observed in the ‘small important improvement group’, which consisted of participants who rated any improvement as ‘a little’, ‘somewhat’, or ‘moderately’ *important* (+ 1 to + 3) on the P-GRC-I scale [51]. Another MIC was calculated using the same method with the T-GRC-I scale. These MIC scores were only considered valid if the ‘small important improvement group’ demonstrated mean changes that were larger than in the ‘not importantly improved’ groups [51] and in samples ≥ 10 participants.

#### MIC: distribution-based methods

##### Half of a standard deviation

Norman et al. [61] proposed the use of 0.5 SD of a sample’s baseline score as a MIC value. We used the SD of the baseline mean scores of the complete sample due to the larger sample size (n = 153).

##### Standard error of measurement

The standard error of measurement (SEM) was taken from the inter-day test–retest reliability analysis based on 65 stable participants of the study cohort who were re-assessed within 1 day [36]. The value of one SEM was taken as the MIC [55].

#### Floor and ceiling effects

For measures with a fixed scale range (DEMMI, HABAM, POMA, SPPB, Barthel Index mobility subscale and FAC), an absolute floor or ceiling effect was considered if > 15% of the participants scored the highest or lowest possible score, respectively [49].

For measures with a ratio unit (4-m gait speed test, 2-min walk test, 5xCRT and TUG), a floor effect was considered if > 15% were not able to perform this measure. An absolute ceiling effect was considered if > 15% of participants reached a score ‘faster/better’ than the normative value for older people (≥ 80 years) ± 1 SD or the upper/lower 95% confidence interval (CI) of the normative value, respectively. We used normative values for women if authors reported sex-stratified values only. The following ceiling effect boarders were used: gait speed = 1.03 m/s (upper 95% CI [62]); 2-min walk test = 142.9 m (upper 95% CI [63]); 5xCRT = 10.7 s (lower 95% CI [64]); TUG = 7.6 s (normative value − 1 SD [65]).

When a patient scores close to one of the extremes, a real change (defined as the minimal detectable change, MDC) could cross that extreme. Patients who score within the MDC-range from one of the extremes can, thus, be regarded as being at either their floor or ceiling as well [66]. Therefore, we additionally calculated floor and ceiling effects related to the MDC-ranges for the extremes. MDC values with 95% confidence of each scale were taken from the reliability analyses based on the same cohort [36]. Admission floor and ceiling effects were calculated based on the baseline sample. Discharge floor and ceiling effects were not calculated due to the small number of participants assessed within 1 week prior to discharge.

## Results

**Fig. 1:**
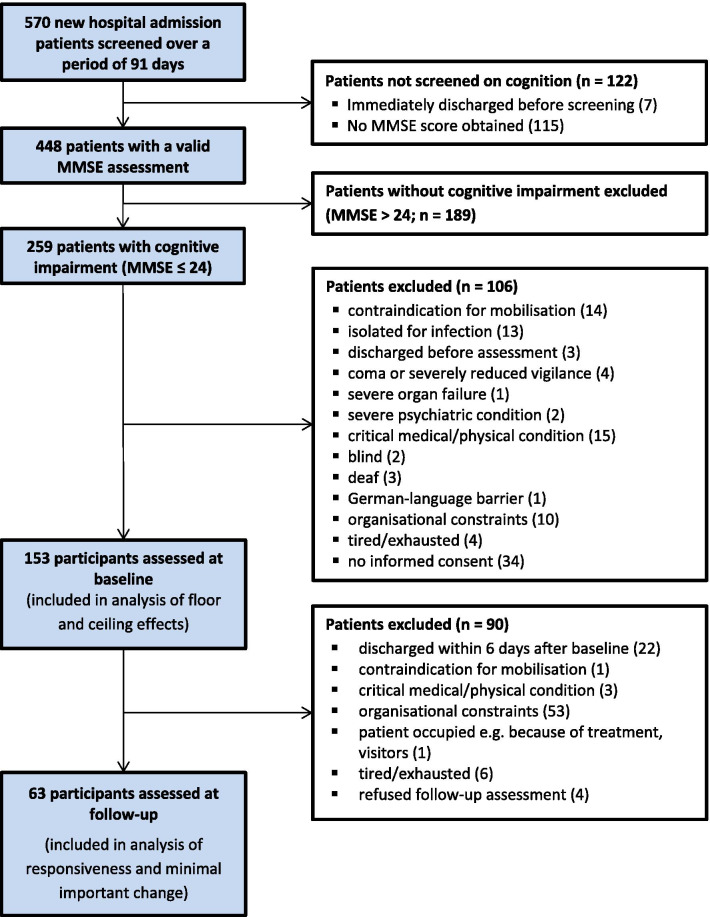
Flow chart of study participants (*MMSE* Mini-Mental State Examination)

A total sample of 63 participants with CSD took part in the follow-up assessment (participant flow: Fig. 1; admission characteristics: Table 2). Study participants included in the follow-up sample (n = 63, 41%) did not differ from participants who did not perform a follow-up measure (n = 90, 59%) with respect to relevant baseline characteristics, such as age, gender or MMSE mean score (see additional results in Additional file 2). However, there were more reports of depression (30% vs 14%) and follow-up participants stayed significantly longer on the acute ward.

**Table 2:** Characteristics of participants at baseline (n = 63)

Characteristic	Value
Age, years	83 ± 6 (69–94)
Gender: male/female, n (%)	39/24 (38/62)
Pre-clinical living situation: home alone/home with family or relatives, n (%)	42/21 (67/33)
Time between admission and baseline assessment, days	3.0 ± 1.5 (0–6)
Time between baseline and follow-up assessment, days	10.8 ± 2.5 (7–17)
Time between follow-up assessment and discharge, days	7.9 ± 8.8 (0–52)
Total length of stay on the acute ward, days	21.8 ± 8.6 (11–64)
Primary diagnosis according to ICD-10 categories	
IX Circulatory, n (%)	11 (17)
X Respiratory, n (%)	6 (10)
XI Digestive, n (%)	4 (6)
XIII Musculoskeletal, n (%)	5 (8)
XVIII Symptoms, signs and abnormal findings, not elsewhere classified, n (%)	8 (13)
XIX Injury, poisoning and certain other consequences of external causes	19 (30)
Other, n (%)	10 (16)
Potential reasons for a cognitive impairment reported in the medical chart (diagnosis, symptom, medical sign; double-counts due to multi-morbidity)	
None reported, n (%)	11 (17)
Alzheimer’s dementia, n (%)	3 (5)
Vascular dementia, n (%)	11 (17)
Dementia, not specified, n (%)	9 (14)
Parkinson’s disease, n (%)	7 (11)
Stroke, n (%)	8 (13)
Depression, n (%)	19 (30)
Delirium, n (%)	15 (24)
Other (psychosis, alcohol abuse), n (%)	5 (8)
Cognitive spectrum disorder	
Dementia alone	16 (25)
Delirium alone	8 (13)
Delirium superimposed on known dementia	7 (11)
Unspecified cognitive impairment^a^	32 (51)
In-hospital walking aid, at baseline/follow-up	
Wheeled-walker or rollator, n (%)	19/24 (30/38)
None, n (%)	12/9 (19/14)
Cane or crutch, n (%)	3/4 (5/6)
Other, n (%)	1/0 (2/0)
Non-ambulatory (wheelchair), n (%)	28/26 (44/41)
Ambulation, at baseline/follow-up	
Independent walkers (FAC ≥ 4), n (%)	18/24 (29/38)
Not ambulatory or dependent walkers (FAC ≤ 3), n (%)	45/39 (71/62)
Barthel Index, 0–100 points, mean score, points	44 ± 20 (0–90)
Mini Mental State Examination, 0–30 points	
Severe cognitive impairment, 0–9 points, n (%)	0 (0)
Moderate cognitive impairment, 10–18 points, n (%)	21 (33)
Mild cognitive impairment, 19–24 points, n (%)	42 (67)
Mean score, points	19.8 ± 3.4 (12–24)
Median score, points	21 (17–23)
Clock Drawing Test, 1–6 points	
Unsuspicious: 1–2 points, n (%)	3 (5)
Suspicious: 3–6 points, n (%)	46 (73)
Missing/not possible, n (%)	1/13 (2/21)
Mean score, points (n = 49)	4.2 ± 1.2 (1–6)
Geriatric Depression Scale short form, 0–15 points	
Normal: 0–4 points, n (%)	28 (44)
Mild depressive: 5–8 points, n (%)	16 (25)
Moderate depressive: 9–11 points, n (%)	9 (14)
Severe depressive: 12–15 points, n (%)	4 (6)
Missing/not possible, n (%)	3/3 (5/5)
Mean score, points (n = 57)	5.5 ± 3.3 (0–13)

Values are presented as mean ± standard deviation (range) or median (interquartile range)

*ICD-10* International Classification of Diseases 10th version, *na* not applicable, *FAC* Functional Ambulation Categories

^a^Mini Mental State Examination score ≤ 24 points, no delirium, no known dementia

A diagnosis of dementia alone was documented in 25% of participants. At baseline, delirium alone was present in 13% participants, 11% of participants had delirium superimposed on dementia and 51% of participants presented with cognitive impairment without documented dementia or delirium. At baseline, according to the MMSE assessment, 33% of participants had a moderate cognitive impairment and 67% had a mild cognitive impairment.

The baseline assessment was performed in the very early phase following hospital admission, within 3 days on average and within 6 days at the most for every participant. The follow-up assessment was performed 10.8 ± 2.5 (range: 7–17) days on average after the baseline assessment and within 7 days prior to discharge for 41 (65%) participants.

Participant performance scores in the 10 mobility measures at baseline and follow-up are given in Table 3 together with respective change scores and effect sizes (small-to-moderate effects).

**Table 3:** Mobility outcome scores of the participants (n = 63)

Measurement instrument	Baseline score [mean ± SD (range)]	Follow-up score [mean ± SD (range)]	Change [mean (95% CI)]	Effect size
de Morton Mobility Index, 0–100 points	35.1 ± 23.1 (0–85)	40.8 ± 22.3 (0–85)	5.7 (3.4 to 7.9)	0.25
Hierarchical Assessment of Balance and Mobility, 0–26 points	12.9 ± 7.9 (0–26)	15.4 ± 7.2 (0–26)	2.5 (1.5 to 3.6)	0.33
Performance Oriented Mobility Assessment, 0–28 points	8.6 ± 9.5 (0–27)	11.0 ± 9.4 (0–28)	2.4 (1.4 to 3.5)	0.25
Short Physical Performance Battery, 0–12 points	2.6 ± 3.5 (0–12)	3.2 ± 3.8 (0–12)	0.6 (0.2 to 1.0)	0.16
4-m gait speed test, m/sec (n = 27)	0.65 ± 0.25 (0.15–1.14)	0.75 ± 0.24 (0.34–1.29)	0.09 (0.04 to 0.15)	0.41
5 × chair rise test, sec (n = 15)	15.8 ± 4.5 (9.2–24.7)	14.6 ± 4.7 (7.2–24.3)	− 1.2 (− 4.0 to 1.5)	0.26
2-min walk test, m (n = 28)	71.8 ± 31.8 (12–126)	82.8 ± 31.8 (19–162)	11.0 (4.6 to 17.5)	0.35
Timed Up and Go test, sec (n = 24)	20.3 ± 8.8 (9.6–48.9)	17.1 ± 5.3 (9.3–30.4)	− 3.3 (− 6.2 to − 0.3)	0.44
Barthel Index mobility subscale, 0–40 points	17.0 ± 13.4 (0–40)	20.7 ± 12.6 (0–40)	3.7 (2.4 to 5.1)	0.28
Functional Ambulation Categories, 0–5 points	1.8 ± 2.0 (0–5)	2.3 ± 2.1 (0–5)	0.5 (0.2 to 0.8)	0.24

*SD* standard deviation,* CI* confidence interval

At baseline, most participants (n = 45, 71%) were not able to walk or needed some kind of assistance for ambulation. This number decreased slightly at follow-up (n = 39, 62%). This resulted in a reduced number of participants available for the responsiveness and MIC analyses at follow-up, as some participants were not able to perform some single-component mobility measures (Table 3; for detailed results, see Additional file 2). The inability to perform these mobility measures was due to insufficient balance, walking, or transfer abilities, or a limited understanding of the test instructions.

The P-GRC-A, P-GRC-I, T-GRC-A, and T-GRC-I scale ratings were available from most patients and therapists, respectively. However, there was substantial disagreement on the amount of change (kappa = 0.47) and the importance of change (kappa = 0.35). Detailed values are presented in the tables in Additional file 2.

### Responsiveness

#### Responsiveness: construct approach

Table 4 provides all correlations between the change scores of each mobility instrument with the change scores of the other instrument scores, and with P-GRC-A and T-GRC-A scale scores. The instruments with the most confirmed hypotheses were the DEMMI (55%) and the FAC (55%), followed by the SPPB (45%), 5xCRT (45%) and the Barthel Index mobility subscale (45%).

**Table 4:** Responsiveness: correlations between change scores of mobility measures with change scores of other mobility measures and with global rating of change scales (n = 63)

	∆ DEMMI	∆ HABAM	∆ POMA	∆ SPPB	∆ Gait speed ^1^†	∆ 5x chair rise test ^2^†	∆ 2-min walk test ^3^†	∆ TUG ^4^	∆ Barthel Index mobility subscale	∆ FAC	P-GRC-A^5^	T-GRC-A^6^	‡
∆ DEMMI		0.67* (0.51 to 0.78)	0.73* (0.59 to 0.83)	0.44* (0.22 to 0.62)	0.24 (− 0.13 to 0.55)	− 0.30 (− 0.67 to 0.19)	0.30 (− 0.06 to 0.59)	− 0.33 (− 0.63 to 0.06)	0.74* (0.60 to 0.83)	0.50* (0.29 to 0.66)	0.40* (0.17 to 0.59)	0.30* (0.05 to 0.51)	6 (55%)
∆ HABAM			0.58* (0.39 to 0.72)	0.20 (− 0.04 to 0.42)	− 0.07 (− 0.42 to 0.3)	0.06 (− 0.42 to 0.51)	0.09 (− 0.27 to 0.43)	− 0.32 (− 0.62 to 0.07)	0.68* (0.52 to 0.79)	0.39* (0.16 to 0.58)	0.23* (− 0.02 to 0.45)	0.34* (0.09 to 0.55)	4 (36%)
∆ POMA				0.46* (0.25 to 0.63)	0.08 (− 0.29 to 0.43)	0.01 (− 0.46 to 0.47)	0.39* (0.04 to 0.65)	− 0.41* (− 0.68 to − 0.04)	0.77* (0.65 to 0.85)	0.60* (0.42 to 0.74)	0.25* (0 to 0.47)	0.28* (0.03 to 0.50)	4 (36%)
∆ SPPB					0.64* (0.36 to 0.81)	− 0.81* (− 0.93 to − 0.55)	0.58* (0.28 to 0.78)	− 0.30 (− 0.61 to 0.09)	0.36* (0.13 to 0.55)	0.55* (0.36 to 0.70)	0.28* (0.04 to 0.49)	0.33* (0.08 to 0.54)	5 (45%)
∆ Gait speed ^1^†						− 0.66* (− 0.86 to − 0.28)	0.67* (0.41 to 0.83)	− 0.47* (− 0.72 to − 0.12)	− 0.02 (− 0.38 to 0.34)	− 0.10 (− 0.44 to 0.27)	0.32 (− 0.05 to 0.61)	0.03 (− 0.33 to 0.39)	4 (36%)
∆ 5x chair rise test ^2^†							− 0.62* (− 0.84 to − 0.22)	0.02 (− 0.45 to 0.48)	− 0.57* (− 0.82 to − 0.14)	− 0.35 (− 0.70 to 0.14)	− 0.49* (− 0.78 to − 0.03)	− 0.15 (− 0.58 to 0.34)	5 (45%)
∆ 2-min walk test ^3^†								− 0.48* (− 0.73 to − 0.12)	0.39* (0.04 to 0.65)	− 0.02 (− 0.37 to 0.34)	0.39* (0.04 to 0.65)	0.12 (− 0.24 to 0.45)	4 (36%)
∆ TUG ^4^									− 0.44* (− 0.7 to − 0.07)	− 0.12 (− 0.48 to 0.27)	− 0.19 (− 0.53 to 0.2)	0.17 (− 0.23 to 0.52)	0 (0%)
∆ Barthel Index mobility subscale										0.52* (0.32 to 0.68)	0.25* (0.00 to 0.47)	0.25* (0.00 to 0.47)	5 (45%)
∆ FAC											0.38* (0.15 to 0.57)	0.33* (0.08 to 0.54)	6 (55%)
P-GRC-A^5^												0.45* (0.22 to 0.63)	NA
T-GRC-A^6^													NA

All correlations are Spearman’s rho with 95% confidence intervals except for correlations between two normally distributed variables, which are Pearson’s r with 95% confidence intervals

*DEMMI* de Morton Mobility Index, *HABAM* Hierarchical Assessment of Balance and Mobility, *POMA* Performance Oriented Mobility Assessment, *SPPB* Short Physical Performance Battery, *TUG* Timed Up and Go test, *FAC* Functional Ambulation Categories, *P-GRC-A* patient-reported amount of mobility change scale, *T-GRC-A* therapist-reported amount of mobility change scale, *NA* not applicable

^∆^change score/delta; ^†^normally distributed data; ^‡^number of confirmed hypotheses

^1^ n = 27; ^2^ n = 15; ^3^ n = 28; ^4^ n = 24; ^5^ n = 61; ^6^ n = 57

^*^Indicates statistical significance (one-sided) with *P* < 0.05

#### Responsiveness: anchor-based approach

The results of anchor-based responsiveness are given in Table 5. The DEMMI was the only instrument with a sufficiently large AUC for all three anchors. The POMA and the 5xCRT had two AUCs ≥ 70% each. The SPPB, 2-min walk test and Barthel Index mobility subscale each showed a sufficiently large AUC with one out of three anchors. The change scores of the HABAM, 4-m gait speed test, TUG, and FAC did not correlate ≥ 0.3 with any anchor or the AUC was under the critical value of 70%.

**Table 5:** Responsiveness of the 10 measurement instruments of mobility (n = 63)

Instrument	Anchor	n	rho	AUC statistics		
				AUC	95% CI	*P*
de Morton Mobility Index	**P-GRC-A**	**58**	**0.40**	**73**	**60–86**	**< 0.01**
	**T-GRC-A**	**55**	**0.35**	**70**	**56–84**	**0.01**
	**FAC-C**	**61**	**0.43**	**76**	**62–90**	**< 0.01**
Hierarchical Assessment of Balance and Mobility	P-GRC-A	58	0.25	64	50–79	0.06
	**T-GRC-A**	**55**	**0.30**	**67**	**52–81**	**0.04**
	FAC-C	61	0.29	68	54–81	0.03
Performance Oriented Mobility Assessment	P-GRC-A	58	0.25	65	50–79	0.06
	**T-GRC-A**	**55**	**0.35**	**70**	**55–85**	**0.01**
	**FAC-C**	**61**	**0.51**	**81**	**68–95**	**< 0.01**
Short Physical Performance Battery	**P-GRC-A**	**58**	**0.32**	**68**	**54–82**	**0.02**
	**T-GRC-A**	**55**	**0.35**	**69**	**55–83**	**0.02**
	**FAC-C**	**61**	**0.51**	**79**	**67–91**	**< 0.01**
4-m gait speed test	P-GRC-A	27	0.24	64	42–86	0.22
	T-GRC-A	25	0.13	57	34–81	0.54
	FAC-C	27	− 0.09	44	22–67	0.64
5 × chair rise test	**P-GRC-A**	**15**	− **0.50**	**80**	**56–100**	**0.06**
	T-GRC-A	14	− 0.14	58	24–93	0.61
	**FAC-C**	**15**	− **0.35**	**73**	**46–99**	**0.19**
2-min walk test	**P-GRC-A**	**28**	**0.42**	**75**	**54–95**	**0.03**
	**T-GRC-A**	**25**	**0.30**	**68**	**46–89**	**0.14**
	FAC-C	28	− 0.03	48	26–70	0.87
Timed Up and Go test	P-GRC-A	24	− 0.09	55	32–79	0.66
	T-GRC-A	22	0.12	43	18–68	0.57
	FAC-C	24	−0.11	57	32–81	0.60
Barthel Index mobility subscale	P-GRC-A	58	0.23	63	49–77	0.09
	T-GRC-A	55	0.27	65	50–79	0.07
	**FAC-C**	**61**	**0.41**	**74**	**60–88**	**< 0.01**
Functional Ambulation Categories	**P-GRC-A**	**58**	**0.36**	**68**	**54–82**	**0.02**
	**T-GRC-A**	**55**	**0.33**	**66**	**51–81**	**0.04**
	FAC-C	na	na	na	na	na

Bold vales are considered valid based on a Spearman’s rho correlation of ≥ 0.3 between the instrument’s change scores and the anchor

*AUC* area under the curve, *P-GRC-A* patient-reported global rating of change amount scale, *T-GRC-A* therapist-reported global rating of change amount scale, *FAC-C* Functional Ambulation Categories change anchor, *na* not applicable

Anchor P-GRC-A: included in analysis (n = 58): unchanged (n = 32), changed/improved (n = 26); excluded (n = 5): deteriorated (n = 3), GRC missing (n = 2)

Anchor T-GRC-A: included in analysis (n = 55): unchanged (n = 29), changed/improved (n = 26); excluded (n = 8): deteriorated (n = 2), GRC missing (n = 6)

Anchor FAC-C: included in analysis (n = 61): unchanged (n = 41), changed/improved (n = 20); excluded (n = 2): deteriorated (n = 2), GRC missing (n = 0)

### Minimal important change (MIC)

For some instruments, the rho correlation between the change scores and the anchor was below the threshold of 0.3 and, therefore, considered invalid (Table 6). Furthermore, there were only four participants in the patient-reported ‘small important improvement group’ (P-GRC-I), so no MIC could be established according to this method.

**Table 6:**
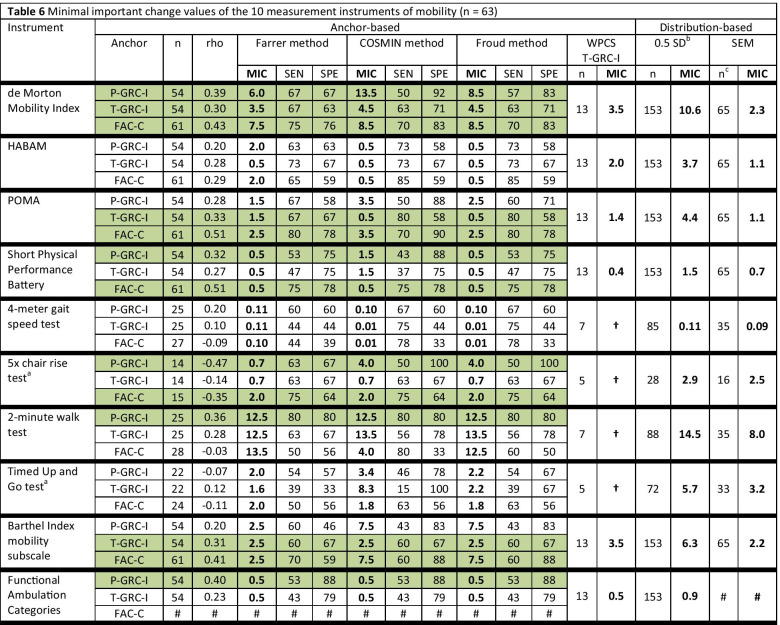
Minimal important change values of the 10 measurement instruments of mobility (n = 63)

Green shaded values indicate ‘valid’ anchor-based MIC values according to Spearman’s rho correlation ≥ 0.3 between the instrument’s change scores and the anchor

*MIC* Minimal important change, *rho* Spearman’s rho correlation coefficient between anchor and instrument’s change score, *COSMIN* COnsensus-based Standards for the selection of health Measurement Instruments, *WPCS* within-patient change score, *SEN* sensitivity, *SPE* specificity, *SD* standard deviation, *SEM* standard error of measurement, *FAC* Functional Ambulation Categories, *HABAM* Hierarchical Assessment of Balance and Mobility, *POMA* Performance Oriented Mobility Assessment, *P-GRC-I* patient-reported global rating of change importance scale, *T-GRC-I* therapist-reported global rating of change importance scale, *FAC-C* Functional Ambulation Categories change anchor

^†^Not calculated because of small sample size (< 10 participants)

^#^FAC-C anchor-based analysis and SEM calculation not possible for the FAC change scores

^a^Change scores for improvement are negative, but MIC values are reported positive

^b^MIC values are based on the baseline sample participants reported in Braun et al. 2018 [35]

^c^MIC values are based on the test–retest reliability sample reported in Braun et al. 2019 [36]

Anchor P-GRC-I: Included in analysis (n = 54): not importantly changed (n = 24), importantly improved (n = 30). Excluded from analysis (n = 9): deteriorated (n = 5), GRC missing (n = 4)

Anchor T-GRC-I: Included in analysis (n = 54): not importantly changed (n = 24), importantly improved (n = 30). Excluded from analysis (n = 9): deteriorated (n = 3), GRC missing (n = 6)

Anchor FAC-C: Included in analysis (n = 61): unchanged (n = 41), changed/improved (n = 20). Excluded from analysis (n = 2): deteriorated (n = 2), GRC missing (n = 0)

**Fig. 2:**
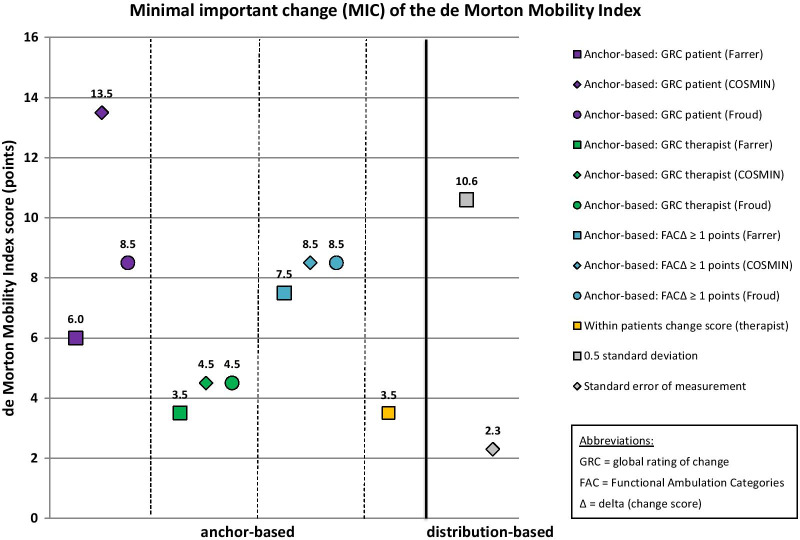
Minimal important change (MIC) values of the de Morton Mobility Index (DEMMI)

**Fig. 3:**
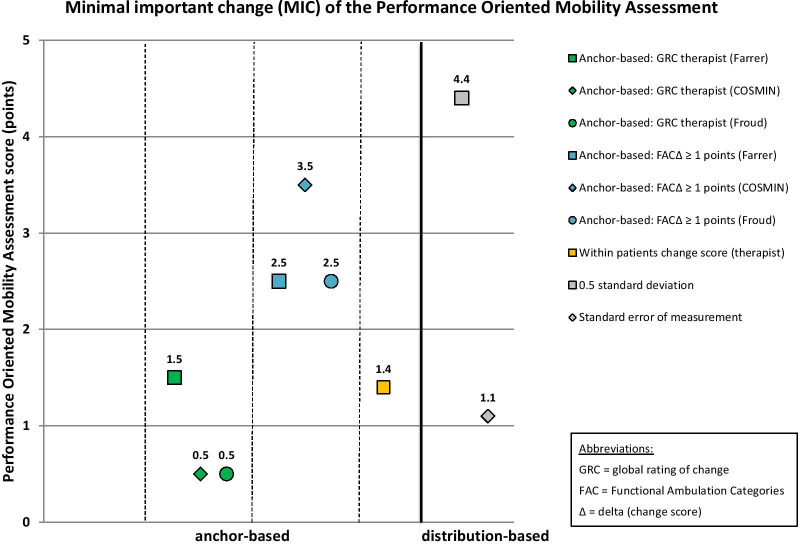
Minimal important change (MIC) values of the Performance-Oriented Mobility Assessment (POMA)

**Fig. 4:**
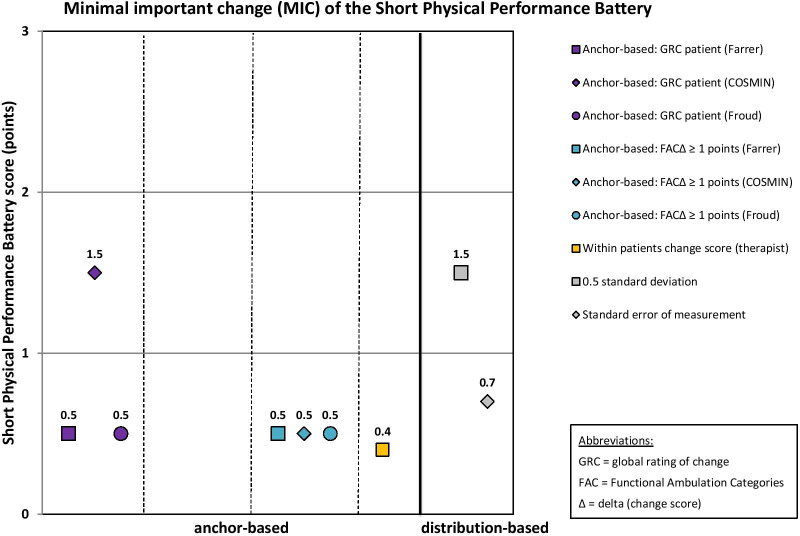
Minimal important change (MIC) values of the Short Physical Performance Battery (SPPB)

**Fig. 5:**
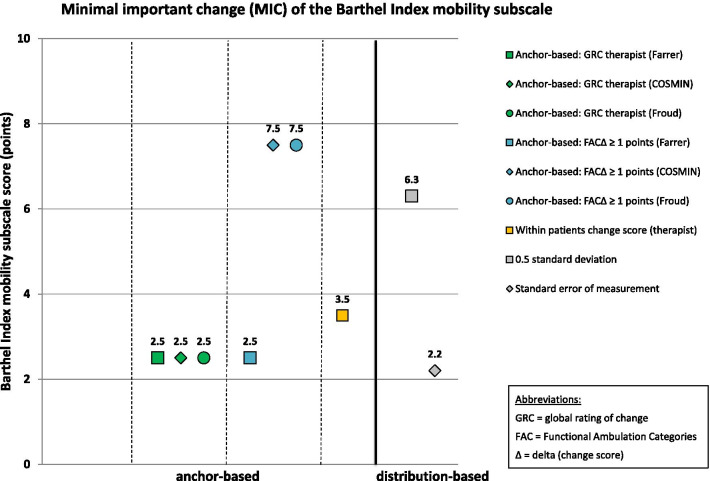
Minimal important change (MIC) values of the Barthel Index mobility subscale

**Fig. 6:**
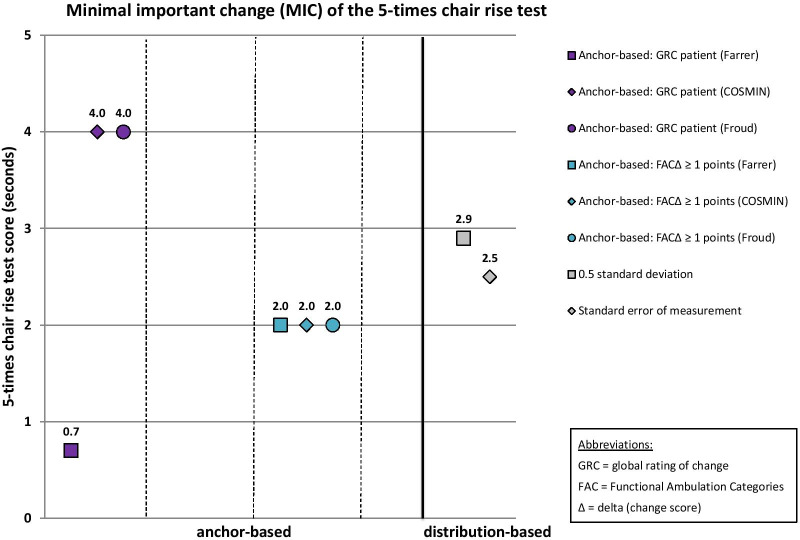
Minimal important change (MIC) values of the 5-times chair rise test (5xCRT)

MIC results of the 10 mobility measures are given in Table 6. MIC values for instruments with rho < 0.3 are reported in this table for the sake of completeness, but these MIC values are considered invalid according to recent beliefs [51]. These values are not illustrated in Figs. 2, 3, 4, 5 and 6, which illustrate MIC values of those measurement instruments with at least five of 10 possible valid anchor-based MIC values (DEMMI, POMA, SPPB, Barthel Index mobility subscale and 5xCRT).

### Floor and ceiling effects

**Fig. 7:**
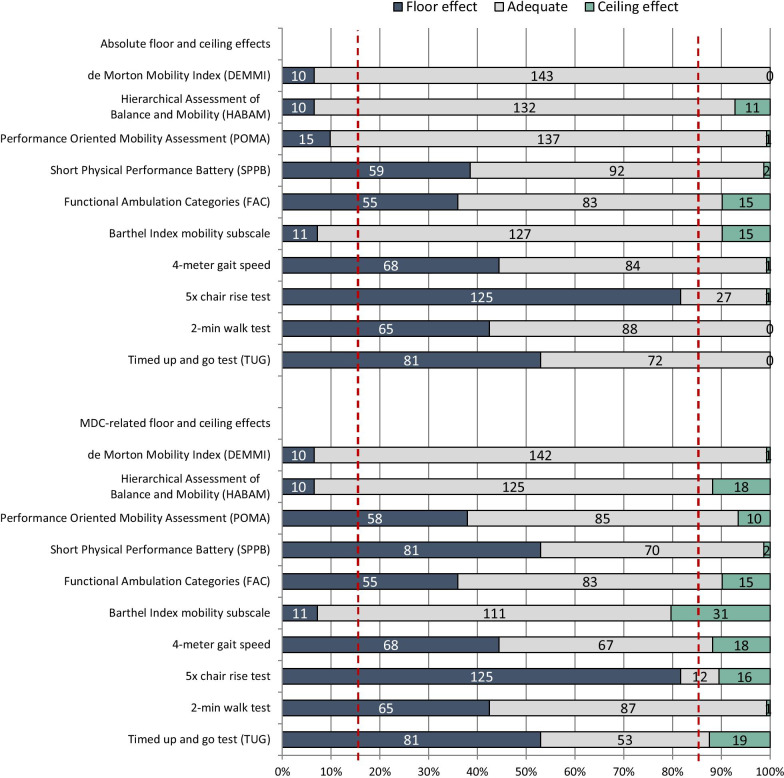
Floor and ceiling effects of mobility measurements at baseline (n = 153). Vertical red dotted lines represent the cut-off value of > 15% for floor and ceiling effects, as proposed by Terwee et al. 2007 [49]

Absolute and MDC-related floor and ceiling effects at baseline (admission) for all mobility measures are given in the table in Additional file 2 and illustrated in Fig. 7.

## Discussion

This is the first study on the responsiveness and interpretability of commonly used measures of mobility in older hospital patients with CSD. The study provides evidence of limited responsiveness for all instruments based on a construct approach. Based on an anchor-based approach, the DEMMI was the only instrument with evidence of sufficient responsiveness and for all other instruments, our analyses provide evidence of limited or insufficient responsiveness. Large floor effects were observed in most instruments. The DEMMI and the HABAM were the only instruments without MDC-related floor and ceiling effects.

### Responsiveness

The DEMMI was the only instrument with an AUC ≥ 0.7 for all three anchors, indicating sufficient responsiveness according to this approach. For five instruments (POMA, 5xCRT, SPPB, 2-min walk test and the Barthel Index mobility subscale) there is conflicting evidence, since these instruments had sufficiently large AUCs in one or two out of three anchors. For the HABAM, 4-m gait speed test, TUG and FAC, there is evidence of no responsiveness, since no AUC was ≥ 0.7, or the change scores did not correlate ≥ 0.3 with any anchor.

According to a construct approach, only two instruments (DEMMI and FAC) had > 50% of confirmed hypotheses (both 55%). No instrument had 75% or more hypotheses confirmed. This threshold has been proposed by the COSMIN group to indicate sufficient responsiveness of a measurement instrument [49, 50]. We recommend interpreting these results with caution, because including the non-responsive instruments (based on the anchor-based approach) as reference instruments in the analyses of responsiveness based on a construct approach might have significantly influenced these analyses.

The comparison of responsiveness approximations found in the present study with existing evidence is limited due to the small number of responsiveness studies performed with older adults with dementia or other cognitive impairments. None of the three psychometric reviews in this field [26, 27, 67] provide any evidence of responsiveness according to an adequate methodology (only effect sizes reported) [49, 50]. Van Iersel et al. [68] assessed the responsiveness of the TUG, the POMA and a short-distance gait speed measure in 85 frail older hospital patients, of whom 45% had dementia. The authors used effect size indices and ROC analyses to assess responsiveness, but did not report AUC values. They concluded that these measures were unsuitable as independent screening instruments for clinically relevant changes in mobility capacity due to the participants’ high intra-individual variability [68]. We are not aware of any other published studies on the responsiveness of mobility measures in older adults with CSD.

According to a recent systematic review on instruments used to evaluate the mobility capacity of older adults during hospitalization [17] and our own literature searches, the responsiveness of the DEMMI has been established with distribution-based methods only and judged as *good* to *excellent* [21, 34, 69]. For the HABAM, responsiveness has not been established so far. In the review [17], responsiveness was judged as *excellent* for the SPPB [70, 71], *good* for the TUG [72], *fair* for the POMA [33], *poor* to *good* for the 6-min walk test [73, 74], and *fair* for gait speed tests [75]. However, most of these studies were performed in non-hospital settings and/or only used methods to assess responsiveness on the basis of effect sizes or other inadequate methods [33, 70, 71, 74, 75]. Thus, results must be interpreted with caution. The comparability of our findings is limited to older hospital patients with CSD.

### Minimal important change

We used anchor-based methods to establish MIC values, with distribution-based MICs as supporting information [51]. We aimed to examine multiple values from different approaches in order to converge on one single value or a small range of values [51].

Anchor-based MIC values for the DEMMI (Fig. 2) range from 3.5 to 13.5, with 9/10 (90%) MIC values ≤ 8.5 points. Thus, we consider a MIC of 9 DEMMI points a robust value, which is 9% of the total DEMMI scale range and close to the MIC of 10 points reported in the DEMMI development study based on a sample of acute older medical patients [21].

We also tried to derive MIC values for the other 9 instruments. A description, based on our study findings, is reported in the Additional file 3. If possible, we also compare our findings to MIC estimations reported in other studies on geriatric patients, taking into account that MIC values are population- and context-specific [58]. The proposed MIC values for each instrument are listed in Table 7.

**Table 7:** Relation between measurement error and minimal important change values of each instrument

Instrument	Measurement error (MDC_90_)^a^	MIC value	MDC_90_ < MIC
de Morton Mobility Index, 0–100 points	5.3	8.5	Yes
Hierarchical Assessment of Balance and Mobility, 0–26 points	2.5	2.0	No
Performance Oriented Mobility Assessment, 0–28 points	2.6	2.5	No
Short Physical Performance Battery, 0–12 points	1.5	0.5	No
4-m gait speed test, m/sec	0.21	0.11	No
5 × chair rise test, sec	5.8	2.0–4.0	No
2-min walk test, m	18.5	12.5	No
Timed Up and Go test, sec	7.4	NA	NA
Barthel Index mobility subscale, 0–40 points	5.1	3.5	No
Functional Ambulation Categories, 0–5 points	NA	0.5	NA

*MDC*_*90*_ minimal detectable change with 90% confidence, *MIC* minimal important change, *NA* not applicable

^a^MDC values are taken from Braun et al. 2019 [36] and are based on the same cohort

### Relating measurement error to the MIC

A measurement instrument should be able to distinguish clinically important change from measurement error. In Table 7, the MIC values from this trial are related to the MDC values with 90% confidence established in the same cohort [36]. According to the COSMIN criteria [50], the DEMMI is the only instrument for which the measurement property ‘measurement error’ can be judged as *good*, since the measurement error is smaller than the MIC.

### Floor and ceiling effects

The clinical value and interpretability of the POMA, SPPB, FAC, 4-m gait speed test, 5xCRT, 2-min walk test and TUG seems considerably limited due to the large MDC-related floor effects, which are evident in 36% (FAC) to 82% (5xCRT) of patients with CSD upon hospital admission. Comparable estimations have been reported for measures of gait and balance that require the patient to stand or walk [8, 11, 32, 34, 35, 76, 77].

Our study underlines that ceiling effects of mobility measures are very unlikely in acute older medical patients with CSD upon hospital admission due to high levels of multimorbidity, frailty and functional impairment.

### Strengths and limitations

This study provides a comprehensive assessment of responsiveness and aspects of interpretability of a broad set of commonly used single- and multi-component performance-based mobility measures in geriatric care. Results allow a head-to-head comparison of these instruments. The selection of instruments was based on psychometric evidence, clinical feasibility, prevalence in the scientific literature and our own awareness [15, 17, 26, 27, 67, 78–80]. Our study includes the most frequently applied instruments in individuals with dementia, such as the TUG, SPPB and 4-m gait speed test [26, 27].

The consecutive baseline sample of 153 participants seems sufficiently large for sound analyses of floor and ceiling effects. The size of the follow-up sample (n = 63) can be judged as *good* according to the COSMIN criteria [14, 46], and baseline characteristics of those participants who did complete a follow-up assessment did not differ from those who did not.

Sampling bias may exist in the data, since the selection of study participants with CSD was based on routine MMSE data [35, 36]. It is possible that we might have missed some potentially eligible patients, because we initially excluded 122 (21%) patients without MMSE assessment. This was caused by organisational constraints, refusal, and vigilance issues, among others. It is not unusual that individuals with CSD refuse cognitive assessment [81, 82]. Thus, we assume that within the group of excluded individuals, there is a significant number of people with (severe) dementia and/or delirium. Further misclassification may be based on participants with intact cognition and depression who scored low on the MMSE [83]. A more detailed, instant and frequent psychiatric review of study participants would have helped to better select and describe the study sample. Further studies should include a more representative sample of patients with a more heterogeneous level of cognitive impairment.

Results of responsiveness are strongly influenced by the validity of the applied methods. A major strength of this study is that we used recommended construct- and anchor-based approaches to establish responsiveness, which are considered more appropriate than responsiveness estimations based on effect size indices [13, 14]. However, the validity of the anchors may be limited. Although global rating of change scales have high face validity [13], the reliability and validity of such retrospective measures of change has been questioned [84, 85]. The trustworthiness of the patient-reported anchor might especially be limited in patients with CSD, of whom many suffer from memory complaints. We also observed that some patients had difficulty in distinguishing between the concepts of *amount* and *importance* of change. Although we provided and accurately explained a broad definition of the concept of mobility, we had the impression that some participants only expressed their impression of change in walking and ambulation. The therapist-reported global rating of change scales may be biased by inaccurate recall of the participants’ baseline mobility capacity in a busy hospital with a large number of different patients. These considerations are underpinned by the low agreement between the patient- and therapist-reported global rating of change scores of ƙ = 0.47 and ƙ = 0.35 for the global rating of mobility change scales of *amount* and *importance*, respectively.

### Implications

The present results are in agreement with our previous findings, indicating that the DEMMI has sufficient measurement properties in terms of feasibility, validity, reliability and responsiveness in older hospital patients with cognitive impairment [35, 36].

Furthermore, the DEMMI was the only instrument that was able to distinguish clinically important change from measurement error in this population. This result has high clinical importance. A healthcare professional who monitors alterations in the mobility capacity of an older patient with CSD must be confident that an observed (meaningful) change in mobility is a true change and not based on measurement error.

Clinicians and researchers can use the MIC values established in this study to plan and evaluate healthcare interventions, for shared decision-making processes, goal setting with patients and relatives, and the planning of sample sizes in clinical trials. However, these MIC values need to be further proven by high-quality, large-scale studies.

For mobility measures that cannot be performed by patients due to functional or cognitive impairments, longitudinal monitoring of mobility is very difficult or impossible. With instruments such as short- and long-distance walk tests, the TUG and chair rise tests, no change scores can be obtained if baseline values or hospital admission test scores are missing. Thus, it is impossible to identify patients who deteriorate or worsen in a mobility capacity by means of these instruments or by any other instrument with large floor effects, such as the POMA, SPPB and FAC. This is of high clinical importance, since mobility measures can be used to identify older patients at high-risk of adverse outcomes. Hubbard et al. [11] reported a relative risk of death for older hospital patients who deteriorated during the first 48 h of admission of 17.1 (95% CI 4.9–60.3) compared to patients whose mobility capacity stabilized or improved. Mobility measures with floor effects seem unsuitable to identify these high-risk patients.

More studies assessing the responsiveness and interpretability of mobility measures in older hospital patients with and without CSD are urgently needed. Furthermore, consensus-based agreement on appropriate methods to determine MIC values is necessary to support authors of psychometric studies in establishing evidence-based MIC values of health-related outcome measures in older people.

## Conclusions

In conclusion, this study provides more evidence for the DEMMI to be a psychometrically sound measurement instrument of mobility in older hospital patients with CSD. The DEMMI has some crucial advantages in comparison to other commonly used instruments, especially concerning its sufficient responsiveness and scale widths. The DEMMI was the only instrument that was able to distinguish clinically important changes from measurement error and has the potential to become the standard measurement instrument of mobility capacity in older hospital patients with CSD.

## Supplementary Information

**Additional file 1.** Detailed description of the assessment procedures and all measurement instruments.

**Additional file 2.** Additional results.

**Additional file 3.** Detailed description and discussion of MIC values of 9 measurement instruments.

## Data Availability

The datasets used and analysed in the current study are available from the corresponding author upon reasonable request.

## Abbreviations

5xCRT: 5-Times chair rise test
AUC: Area under the receiver operating characteristic curve
CI: Confidence interval
COSMIN: COnsensus-based Standards for the selection of health Measurement Instruments
CSD: Cognitive spectrum disorders
DEMMI: De Morton Mobility Index
FAC: Functional Ambulation Categories
HABAM: Hierarchical Assessment of Balance and Mobility
MDC: Minimal detectable change
MIC: Minimal important change
MMSE: Mini-Mental State Examination
P-GRC-A: Patient-reported global rating of change amount scale
P-GRC-I: Patient-reported global rating of change importance scale
POMA: Performance Oriented Mobility Assessment
SEM: Standard error of measurement
SD: Standard deviation
SPPB: Short Physical Performance Battery
T-GRC-A: Therapist-reported global rating of change amount scale
T-GRC-I: Therapist-reported global rating of change importance scale
TUG: Timed Up and Go test
