# Supplementary material for: Responsiveness and interpretability of commonly used outcome assessments of mobility capacity in older hospital patients with cognitive spectrum disorders
Source: Health Qual Life Outcomes. 2021 Mar 1;19:68. doi: 10.1186/s12955-021-01690-3 (PMC7923341; doi:10.1186/s12955-021-01690-3)
Supplement: Supplementary file 1 — Additional file 1. Detailed description of the assessment procedures and all measurement instruments. [file 12955_2021_1690_MOESM1_ESM.pdf]

Additional file 1: Detailed description of the assessment procedures and all measurement instruments

All study assessments were performed in the patient's hospital room and on the ward. Whenever a participant had difficulty understanding the instructions of any assessment, the examiner was allowed to demonstrate individual items or tasks, use verbal persuasion, physical cueing and physical assistance, but only if this was allowed according to the official instrument instructions. Breaks were allowed between assessments. A hand-held digital stopwatch was used for all temporal outcomes. Walking distances were recorded with a digital measuring wheel.

Patients were scored at their highest level of safe function, using their usual walking aid. The same device was used for all assessments in a single session. Similar items in different assessments were only performed once to reduce participant's burden, e.g. standing with both feet together is required in the DEMMI and the Performance Oriented Mobility Assessment (POMA). For participants requiring some kind of physical assistance during ambulation (Functional Ambulation Categories score ≤2), the walking tests were scored as "unable" (timed up and go test, gait speed, 2 minute walk test) or "0 points" (POMA gait subscale), respectively.

We used a broad set of clinically established comparator instruments that show sufficient validity and reliability on older people. However, the measurement properties of most comparator instruments in older people with cognitive impairment are unclear since they have never, or only insufficiently been analysed.

The **Hierarchical Assessment of Balance and Mobility (HABAM)** is a clinical bedside and interval level mobility assessment that quantifies functional abilities in the sub-categories balance, transfers and ambulation. Higher scores indicate higher ability (0 to 26 points). There are no reports of the HABAM's psychometrics in older people with cognitive impairment, but multiple studies indicate sufficient reliability, content and construct validity in various populations of older people.[1–3]

The **Performance Oriented Mobility Assessment (POMA)** is a well-established clinician-rated measure of older people's mobility and fall risk [4]. The maximum ordinal score of 28 points indicates better mobility. The POMA has sufficient reliability [5, 6] and predictive validity [5] in older people with moderate to severe dementia, but significant feasibility problems have also been reported for this population [5].

The clinician-completed **Functional Ambulation Categories (FAC)** distinguishes 6 levels of walking ability subjected to the amount of assistance required over a walking distance of 10 meters [7]. Lower

scores, where physical assistance is needed, indicate poorer mobility than higher scores, where the patient is able to ambulate independently. The FAC has never been analysed in people with dementia but adequate predictive validity as well as excellent concurrent validity and reliability have been reported for patients with stroke [8].

The **2-minute walk test (2minWT)** can be used to quantify walking endurance and functional-exercise capacity [9]. By measuring similar constructs, the 2minWT seems to be better tolerated by geriatric inpatients than the 6 minute walk test [10]. Subjects were asked to walk as far as possible within two minutes on the hospital corridor. Only one trial was performed to avoid fatigue effects. The psychometric properties in people with Alzheimer's dementia have not been established for the 2minWT but for the 6 minute walk test, which seems quite comparable and shows sufficient reproducibility [11–13].

The **Short Physical Performance Battery (SPPB)** is a measure of mobility and physical functioning. It includes three objective tests of lower body functions: a hierarchical test of standing balance, a four meter walk test (4mWT) and five times chair rise test (5xCRT) [14]. The SPPB has a scoring from 0 (unable) to 4 points for every sub-test, with a maximum of 12 points in total (ordinal scaled). Sufficient validity and reliability of the SPPB have been described for older people with and without dementia [14–16].

Habitual **gait speed** in m/s over a distance of four meter was assessed as part of the SPPB. Timing was started when the participant began walking. The shorter time of two trials was used for analysis [17]. Walking speed is an objective and reliable physical performance test to evaluate functional capacity of the lower limbs and mobility with well-documented predictive value for major health-related outcomes in older people with and without dementia [11, 12, 16, 18, 19].

For the **5 times chair rise test (5xCRT)**, participants were asked to stand up and sit down from a chair as fast as possible for five times, with the hands being crossed in front of the chest. Shorter times indicate higher mobility. The 5xCRT seems to be a reproducible assessment in people with dementia [11, 20, 21].

The **Timed Up and Go test (TUG)** is a performance based test that assesses basic mobility functions. The patient is asked to stand up from a chair, walk 3 meter, turn around, walk back and return to the chair [22]. In the present study, chair height was 46 cm, the participant was placed with the trunk leant backwards, the arms rested on the armrest and a cone had to be encircled. Participants chose the turning side. A familiarization trial was followed by two counted trials, of which the mean (in sec) was the final TUG score. At least one counted trial must have been valid to be included in the analysis. Shorter times indicate higher mobility. There is conflicting evidence for the TUG to be a sufficiently

reliable test in people with dementia [11, 12, 20, 21, 23], the construct validity has not been examined properly and feasibility limitations have been reported due to significant floor effects [23].

The **Barthel Index** [24] (BI; 0-100 points) is a performance based measure of functioning and independence in the activities of daily living (ADL). Higher scores indicate better functioning. The Barthel Index has been reported to be the most widely used measure of ADL function [25]. In this study, the BI was applied by the nursing staff as part of routine care within the first 7 days after admission. Since most of these scores were recorded in a considerable time frame from the DEMMI scores, the BI score was only used to describe the sample. For the psychometric analysis, we re-assessed the 3 mobility items #*transfer* (0 to 15 points), #*walking* (0 to 15 points) and #*climbing stairs* (0 to 10 points) [26] in the study assessment session. We summed these items to a Barthel Index mobility subscale (0 to 40 points). This subscale has sufficient face validity and the reliability of these 3 single BI items has been reported to be fair to excellent in various studies including individuals with stroke [27] and older people with and without cognitive impairment [28].

The **Mini Mental State Examination Test (MMSE)** [29] is an 11-item assessment of cognitive function that assesses orientation, registration, attention or calculation (serial sevens or spelling), recall, naming, repetition, comprehension (verbal and written), writing, and construction. Scores can range from 1 to 30. By convention, scores <24 points indicate increasing cognitive impairment [30, 31]. The cognitive impairment can be judged as severe (≤9 points), moderate (10–18 points) or mild (19–23 points) according to the MMSE score, although other cut-off points have been suggested due to the wide spectrum in the severity of disease that people with dementia have [32, 33].

The **Clock Drawing Test (CDT)** [34] is one of the most widely used cognitive screening tools to measure a variety of cognitive functions, including selective and sustained attention, auditory comprehension, verbal working memory, numerical knowledge, visual memory and reconstruction, visuospatial abilities and executive function. There are multiple CDT administration and scoring systems [35]. The study hospital used a 6-point scoring system, with higher scores reflecting a higher number of errors and more cognitive impairment [36]. Scores ≥3 points are considered indicative of cognitive dysfunction.

The short version of the **Geriatric Depression Scale (GDS-15)** is a 15-item dichotomous patient-report outcome measure used to identify depression in older people [37, 38]. The severity of depressive symptoms can be judged as normal (0-4 points), mildly (5-8 points), moderately (9-11 points) and severely depressed (12-15 points) [38].

References Additional file 1

1. MacKnight C, Rockwood K. Rasch analysis of the hierarchical assessment of balance and mobility (HABAM). J Clin Epidemiol. 2000;53:1242–7.

2. Braun T, Rieckmann A, Grüneberg C, Marks D, Thiel C. Hierarchical assessment of balance and mobility. Zeitschrift für Gerontologie und Geriatrie. 2016;49:386–97.
3. Rockwood K, Rockwood MRH, Andrew MK, Mitnitski A. Reliability of the hierarchical assessment of balance and mobility in frail older adults. J Am Geriatr Soc. 2008;56:1213–7.
4. Tinetti ME. Performance-oriented assessment of mobility problems in elderly patients. J Am Geriatr Soc. 1986;34:119–26.
5. Sterke CS, Huisman SL, van Beeck EF, Looman CWN, van der Cammen TJM. Is the Tinetti Performance Oriented Mobility Assessment (POMA) a feasible and valid predictor of short-term fall risk in nursing home residents with dementia? Int Psychogeriatr. 2010;22:254–63.
6. van Iersel MB, Benraad CEM, Rikkert MGMO. Validity and reliability of quantitative gait analysis in geriatric patients with and without dementia. J Am Geriatr Soc. 2007;55:632–4.
7. Holden MK, Gill KM, Magliozzi MR, Nathan J, Piehl-Baker L. Clinical gait assessment in the neurologically impaired. Reliability and meaningfulness. Phys Ther. 1984;64:35–40.
8. Mehrholz J, Wagner K, Rutte K, Meissner D, Pohl M. Predictive validity and responsiveness of the functional ambulation category in hemiparetic patients after stroke. Arch Phys Med Rehabil. 2007;88:1314–9.
9. Pin TW. Psychometric Properties of 2-Minute Walk Test: A Systematic Review. Arch Phys Med Rehabil;95:1759–75.
10. Brooks D, Davis AM, Naglie G. The feasibility of six-minute and two-minute walk tests in in-patient geriatric rehabilitation. Can J Aging. 2007;26:159–62.
11. Blankevoort CG, van Heuvelen MJG, Scherder EJA. Reliability of Six Physical Performance Tests in Older People With Dementia. Phys Ther. 2013;93:69–78.
12. Ries JD, Echternach JL, Nof L, Gagnon Blodgett M. Test-retest reliability and minimal detectable change scores for the timed "up & go" test, the six-minute walk test, and gait speed in people with Alzheimer disease. Phys Ther. 2009;89:569–79.
13. Tappen RM, Roach KE, Buchner D, Barry C, Edelstein J. Reliability of physical performance measures in nursing home residents with Alzheimer's disease. J. Gerontol. A Biol. Sci. Med. Sci. 1997;52:5.
14. Guralnik JM, Simonsick EM, Ferrucci L, Glynn RJ, Berkman LF, Blazer DG, et al. A short physical performance battery assessing lower extremity function: association with self-reported disability and prediction of mortality and nursing home admission. J Gerontol. 1994;49:85–94.
15. Guralnik JM, Ferrucci L, Simonsick EM, Salive ME, Wallace RB. Lower-extremity function in persons over the age of 70 years as a predictor of subsequent disability. N. Engl. J. Med. 1995;332:556–61.

16. Fox B, Henwood T, Neville C, Keogh J. Relative and absolute reliability of functional performance measures for adults with dementia living in residential aged care. Int Psychogeriatr. 2014;26:1659–67.
17. Graham JE, Ostir GV, Fisher SR, Ottenbacher KJ. Assessing walking speed in clinical research: a systematic review. J Eval Clin Pract. 2008;14:552–62.
18. Muñoz Mendoza C, Cabrero García J, Reig Ferrer A, Cabañero Martínez MJ. Evaluation of walking speed tests as a measurement of functional limitations in elderly people: A structured review. International Journal of Clinical and Health Psychology. 2010;10:359–78.
19. Peel NM, Kuys SS, Klein K. Gait speed as a measure in geriatric assessment in clinical settings: a systematic review. J Gerontol A Biol Sci Med Sci. 2013;68:39–46.
20. Suttanon P, Hill KD, Dodd KJ, Said CM. Retest reliability of balance and mobility measurements in people with mild to moderate Alzheimer's disease. Int. Psychogeriatr. 2011;23:1152–9.
21. Thomas VS, Hageman PA. A preliminary study on the reliability of physical performance measures in older day-care center clients with dementia. Int Psychogeriatr. 2002;14:17–23.
22. Podsiadlo D, Richardson S. The timed "Up & Go": a test of basic functional mobility for frail elderly persons. J Am Geriatr Soc. 1991;39:142–8.
23. Rockwood K, Awalt E, Carver D, MacKnight C. Feasibility and measurement properties of the functional reach and the timed up and go tests in the Canadian study of health and aging. J. Gerontol. A Biol. Sci. Med. Sci. 2000;55:3.
24. Mahoney FI, Barthel DW. Functional Evaluation: The Barthel Index. Md State Med J. 1965;14:61–5.
25. Wade DT. Measurement in neurological rehabilitation. Curr Opin Neurol Neurosurg. 1992;5:682–6.
26. Lubke N, Meinck M, Renteln-Kruse W von. The Barthel Index in geriatrics. A context analysis for the Hamburg Classification Manual. Z Gerontol Geriatr. 2004;37:316–26.
27. Duffy L, Gajree S, Langhorne P, Stott DJ, Quinn TJ. Reliability (inter-rater agreement) of the Barthel Index for assessment of stroke survivors: systematic review and meta-analysis. Stroke. 2013;44:462–8.
28. Sainsbury A, Seebass G, Bansal A, Young JB. Reliability of the Barthel Index when used with older people. Age Ageing. 2005;34:228–32.
29. Folstein MF, Folstein SE, McHugh PR. "Mini-mental state". A practical method for grading the cognitive state of patients for the clinician. J Psychiatr Res. 1975;12:189–98.
30. Mitchell AJ. A meta-analysis of the accuracy of the mini-mental state examination in the detection of dementia and mild cognitive impairment. J Psychiatr Res. 2009;43:411–31.
31. Mungas D. In-office mental status testing: a practical guide. Geriatrics. 1991;46:54-8, 63, 66.

32. Crum RM, Anthony JC, Bassett SS, Folstein MF. Population-Based Norms for the Mini-Mental State Examination by Age and Educational Level. JAMA. 1993;269:2386–91.
33. Kukull WA, Larson EB, Teri L, Bowen J, McCormick W, Pfanschmidt ML. The mini-mental state examination score and the clinical diagnosis of dementia. Journal of Clinical Epidemiology. 1994;47:1061–7.
34. Shulman KI. Clock-drawing: is it the ideal cognitive screening test? Int J Geriatr Psychiatry. 2000;15:548–61.
35. Mainland BJ, Amodeo S, Shulman KI. Multiple clock drawing scoring systems: simpler is better. Int J Geriatr Psychiatry. 2014;29:127–36.
36. Shulman KI, Pushkar Gold D, Cohen CA, Zucchero CA. Clock-drawing and dementia in the community: A longitudinal study. Int. J. Geriat. Psychiatry. 1993;8:487–96.
37. Yesavage JA, Sheikh JI. Geriatric Depression Scale (GDS) - Recent Evidence and Development of a Shorter Version. Clinical Gerontologist. 2008;5:165–73.
38. Greenberg SA. How to try this: the Geriatric Depression Scale: Short Form. Am J Nurs. 2007;107:60-9; quiz 69-70.
