# Supplementary material for: Responsiveness and interpretability of commonly used outcome assessments of mobility capacity in older hospital patients with cognitive spectrum disorders
Source: Health Qual Life Outcomes. 2021 Mar 1;19:68. doi: 10.1186/s12955-021-01690-3 (PMC7923341; doi:10.1186/s12955-021-01690-3)
Supplement: Supplementary file 2 — Additional file 2. Additional results. [file 12955_2021_1690_MOESM2_ESM.pdf]

Additional file 2: Additional results

Comparison of participants' characteristics between the study sample (n = 63) and excluded participants (n = 90)

Characteristic		Values by sample		P
		Included (n = 63)	Excluded (n = 90)	
**Age, years**		83 ± 6	81.9 ± 7.1	0.57
**Gender: male/female, n (%)**		39/24 (38/62)	30/60 (33/67)	0.54
**Total length of stay on the acute ward, days**		21.8 ± 8.6	15.9 ± 6.6	<0.01
**Time between admission and assessment, days**		3.0 ± 1.5 (0 – 6)	3.1 ± 1.6	0.88
**Primary diagnosis according to ICD-10 categories**				
	IX Circulatory, n (%)	11 (17)	13 (14)	0.95
	X Respiratory, n (%)	6 (10)	8 (9)	
	XI Digestive system, n (%)	4 (6)	3 (3)	
	XIII Musculoskeletal, n (%)	5 (8)	7 (8)	
	XVIII Symptoms, signs and abnormal clinical and laboratory findings, not elsewhere classified, n (%)	8 (13)	10 (11)	
	XIX Injury, poisoning and certain other consequences of external causes, n (%)	19 (30)	33 (37)	
	Other, n (%)	10 (16)	16 (18)	
**Potential reasons for cognitive impairment reported in the medical chart (diagnosis, symptom, medical sign; double-counts possible due to multi-morbidity)**				
	None reported, n	11 (17)	38 (42)	<0.01
	Alzheimer's dementia, n	3 (5)	4 (4)	0.92
	Vascular dementia, n	11 (17)	13 (14)	0.61
	Frontotemporal dementia, n	0	1 (1)	na
	Dementia, not specified, n	9 (14)	20 (22)	0.22
	Dementia, any kind, n	23 (37)	38 (42)	0.48
	Parkinson's disease, n	7 (11)	5 (6)	0.21
	Stroke, n	8 (13)	10 (11)	0.76
	Depression, n	19 (30)	13 (14)	0.02
	Delirium, n	15 (24)	14 (14)	0.14
	Other (psychosis, alcohol abuse, Vitamin B6 deficiency), n	5 (8)	3 (3)	0.21
**Cognitive spectrum disorder**				
	Dementia alone	16 (25)	29 (32)	0.38
	Delirium alone	8 (13)	5 (6)	
	Delirium superimposed on known dementia	7 (11)	8 (9)	
	Unspecified cognitive impairment [a]	32 (51)	48 (53)	
**In-hospital walking aid**				
	None, n (%)	12 (19)	10 (11)	0.10
	Walking aid: e.g. wheeled-walker, cane, n (%)	23 (37)	48 (53)	
	Non-ambulatory (wheelchair), n (%)	28 (44)	32 (36)	
**Ambulation**				
	Independent walkers (FAC ≥ 4), n (%)	18 (29)	25 (39)	0.91
	Not ambulatory or dependent walkers (FAC ≤ 3), n (%)	45 (71)	65 (61)	
**Barthel Index, 0 – 100 points**				
	Mean score, points (n = 148)	44 ± 20 (n = 63)	46 ± 20 (n = 85)	0.49
**Mini Mental State Examination, 0 – 30 points**				
	Mean score, points	19.8 ± 3.4 (12 – 24)	18.9 ± 4.2	0.14
**Clock Drawing Test, 1 – 6 points**				
	Mean score, points (n = 122)	4.2 ± 1.2 (n = 49)	4.3 ± 1.4 (n = 73)	0.79
**Geriatric Depression Scale short form, 0 – 15 points**				
	Mean score, points (n = 135)	5.5 ± 3.3 (n = 57)	4.9 ± 2.9	0.26

Abbreviations: ICD-10 = International Classification of Diseases 10th version; na = not applicable; FAC = Functional Ambulation Categories
[a] Mini Mental State Examination score ≤24 points, no delirium, no known dementia
Values are presented as mean ± standard deviation (range) or median (interquartile range).

Floor effects of four mobility measures (assessment administration is not possible)

		Follow-up assessment		n
		possible	not possible	
**4-meter gait speed test**				
Baseline assessment	possible	27	1	28
	not possible	7	28	35
		34	29	63
**5x chair rise test**				
Baseline assessment	possible	15	0	15
	not possible	1	47	48
		16	47	63
**2-minute walk test**				
Baseline assessment	possible	28	2	30
	not possible	7	26	33
		35	28	63
**Timed Up and Go test**				
Baseline assessment	possible	24	0	24
	not possible	7	32	39
		31	32	63

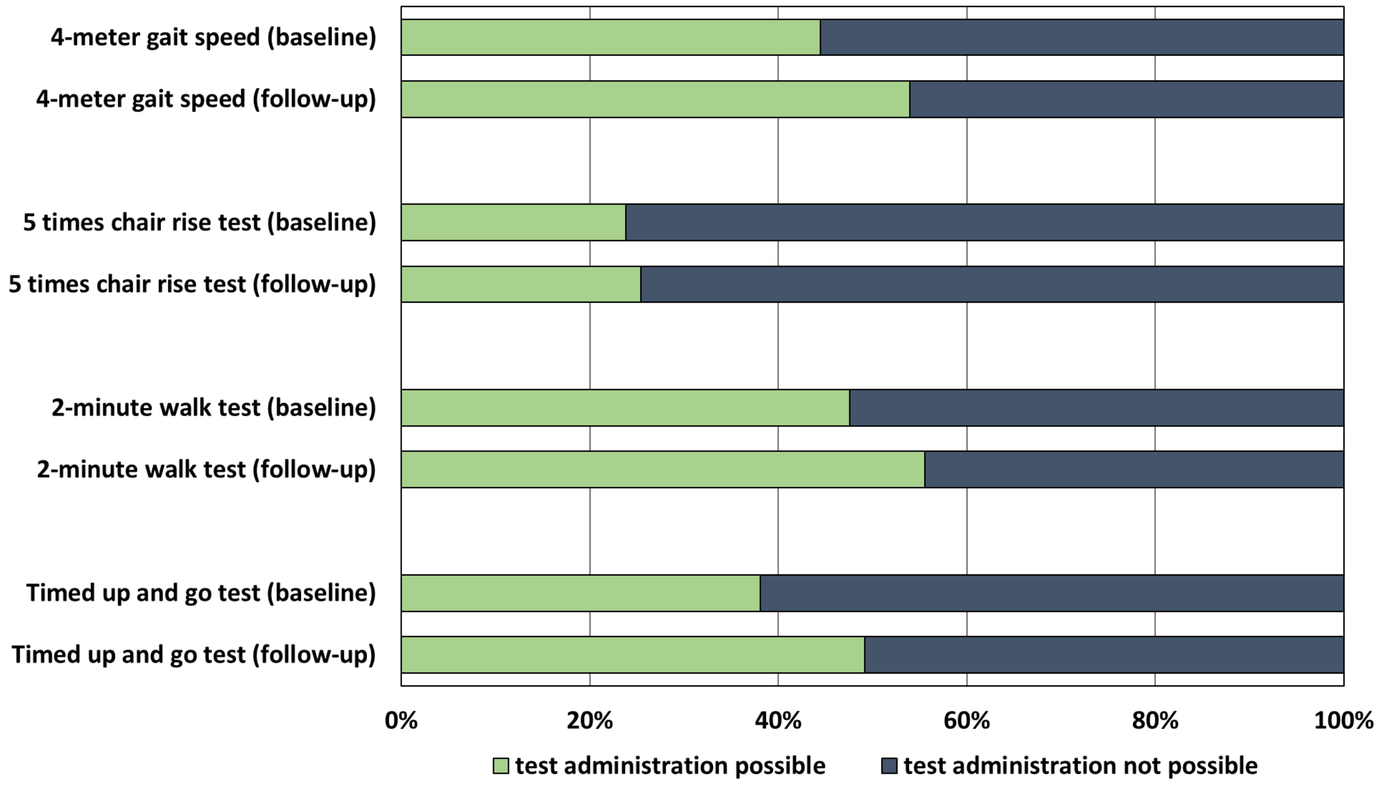

Agreement in the amount and importance of mobility change reported by the patients and by the therapists

**Agreement in the AMOUNT of mobility change by patients and therapists**						
Cohen's kappa with linear weighting (n = 55): 0.47 (95% CI: 0.29 to 0.66)		Therapist-reported global rating of change (physiotherapist: n = 48; occupational therapist: n = 9)				
		Improved (T-GRC-A ≥ +2)	Unchanged (T-GRC-A = -1 to +1)	Deteriorated (T-GRC-A ≤ -2)	†	Total n
Patient-reported global rating of change	Improved (P-GRC-A ≥ +2)	21	7	0	4	32
	Unchanged (P-GRC-A = -1 to +1)	5	17	2	2	26
	Deteriorated (P-GRC-A ≤ -2)	0	3	0	0	3
	‡	0	2	0	0	2
	Total n	26	29	2	6	63

P-GRC-A = patient-reported amount of change (global rating of mobility change)
T-GRC-A = therapist-reported amount of change (global rating of mobility change)
CI = confidence interval

† = missing: therapist change over hospital stay (n = 6)
‡ = missing: patient did not understand the concept of global rating of change / global transition question (n = 2)

**Agreement in the IMPORTANCE of mobility change by patients and therapists**						
Cohen's kappa with linear weighting (n = 54): 0.35 (95% CI: 0.16 to 0.53)		Therapist-reported global rating of change (physiotherapist: n = 48; occupational therapist: n = 9)				
		Importantly improved (T-GRC-I ≥ +1)	Not importantly improved (T-GRC-I = 0)	Deteriorated	†	Total n
Patient-reported global rating of change	Importantly improved (P-GRC-I ≥ +1)	21	6	0	3	30
	Not importantly improved (P-GRC-I = 0)	9	11	2	2	24
	Deteriorated	0	5	0	0	5
	‡	0	2	1	1	4
	Total n	30	24	3	6	63

P-GRC-I = patient-reported importance of change (global rating of mobility change)
T-GRC-I = therapist-reported importance of change (global rating of mobility change)
CI = confidence interval

† = missing: therapist change over hospital stay (n = 6)
‡ = missing: patient did not understand the concept of global rating of change / global transition question (n = 4)

Absolute and MDC-related floor and ceiling effects at baseline (n = 153)

Outcome Assessment	Scale width	Absolute floor and ceiling		MDC-related floor and ceiling				
					MDC_{95}-range from		% of patients scoring within MDC_{95}-range	
		Floor	Ceiling	MDC_{95}	Floor	Ceiling	Floor	Ceiling
DEMMI	0 – 100 points*	10 (7%)	0 (0%)	6.3	0 – 6	84 – 100	10 (7%)	1 (1%)
HABAM	0 – 26 points*	10 (7%)	11 (7%)	2.9	0 – 2	24 – 26	10 (7%)	18 (12%)
POMA	0 – 28 points*	15 (10%)	1 (1%)	3.1	0 – 3	25 – 28	58 (38%)	10 (7%)
SPPB	0 – 12 points*	59 (39%)	2 (1%)	1.9	0 – 1	11 – 12	81 (53%)	2 (1%)
FAC	0 – 5 Points*	55 (36%)	15 (10%)	na	0	5	55 (36%)	15 (10%)
BI mobility subscale	0 – 40 points*	11 (7%)	15 (10%)	6.1	0 – 6	34 – 40	11 (7%)	31 (20%)
Gait speed	Ratio scale (meter/second)	68 (44%)	1 (1%)	0.25	UAT	0.78 – 1.03	68 (44%)	18 (12%)
5x chair rise test	Ratio scale (seconds)	125 (82%)	1 (1%)	6.9	UAT	10.7 – 17.6	125 (82%)	16 (10%)
2-min walk test	Ratio scale (meter)	65 (42%)	0 (0%)	22.1	UAT	120.8 – 142.9	65 (42%)	1 (1%)
TUG	Ratio scale (seconds)	81 (53%)	0 (0%)	8.8	UAT	7.6 – 16.4	81 (53%)	19 (12%)

Abbreviations: MDC = minimal detectable change; DEMMI = de Morton Mobility Index; HABAM = Hierarchical Assessment of Balance and Mobility; POMA = Performance Oriented Mobility Assessment; SPPB = Short Physical Performance Battery; FAC = Functional Ambulation Categories; BI = Barthel Index; TUG = timed up and go test; na = not applicable; UAT = unable to perform.

From left to right, for all scales, the absolute scale width and the absolute floor and ceiling effects are presented. Followed by the minimal detectable change (MDC_{95}) and the percentage of scores that fell within the MDC_{95}-range for both extremes.

*Ranges reflect lower to high mobility functions.

Absolute ceiling boarders for ratio scales based on normative values: gait speed: 1.03 m/s; 5xCRT = 10.7 seconds; 2-min walk test = 142.9 m; TUG = 7.6 seconds.
