# Supplementary material for: Responsiveness and interpretability of commonly used outcome assessments of mobility capacity in older hospital patients with cognitive spectrum disorders
Source: Health Qual Life Outcomes. 2021 Mar 1;19:68. doi: 10.1186/s12955-021-01690-3 (PMC7923341; doi:10.1186/s12955-021-01690-3)
Supplement: Supplementary file 3 — Additional file 3. Detailed description and discussion of MIC values of 9 measurement instruments. [file 12955_2021_1690_MOESM3_ESM.pdf]

Additional file 3: Detailed description and discussion of MIC values of 9 measurement instruments

For the HABAM, no MIC values have been established yet [1, 2], and it was not possible to establish valid MIC values for the HABAM based on an anchor-based approach in the present study. However, the correlations between the HABAM change scores and the three anchors were between 0.20 and 0.29, thus, slightly below the 0.3 cut-off. Taking into account the distribution-based MIC values as well, we consider 2 points (8% of the scale width) a potential MIC estimation for the HABAM in patients with CSD, but this needs to be confirmed by further investigations.

POMA anchor-based MIC values range from 0.5 to 3.5 (Figure 3), with 6/7 (86%) MIC values ≤ 2.5 points. Thus, a MIC of 3 points for the POMA seems to be a robust estimation (11% of the scale width). We could not find any reports on MIC estimations for the POMA in older people.

For the SPPB, anchor-based MIC values range from 0.4 to 1.5 (Figure 4), with 6/7 (86%) MIC values ≤ 0.5 points. Thus, a change in the SPPB of ≥ 1 point seems to be clinically relevant (8% of the 12-point scale). Perera et al. [3] reported small meaningful change estimates for the SPPB from 0.27 to 0.55 points in samples of community-dwelling older adults and sub-acute stroke survivors, indicating a MIC of 1 point as well.

For short-distance gait speed, we could not establish any valid MIC estimations. However, as presented in Table 6, values range from 0.01 to 0.11 m/s. In a systematic review, Bohannon et al. [4] analysed gait speed MIC values reported in seven articles, including people with stroke, hip fracture, multiple sclerosis, or mixed pathologies. The authors concluded that changes in gait speed of 0.10 to 0.20 m/s may be important across multiple patient groups. For older hospital patients with CSD, improvements in gait speed of 0.11 m/s or more may be clinically relevant (17% of baseline mean score), but this MIC estimation needs to be proven further.

For the 5xCRT, 4/6 (67%) anchor-based MIC values were ≤ 2.0 seconds (Figure 6). We could not find any MIC estimations for older patients in the literature. Meretta et al. [5] reported a MIC of 2.3 seconds for 117 patients with vestibular disorders (mean age: 63 years). A MIC between 2.0 and 4.0 seconds may be a valid estimation in older hospital patients with CSD (13% and 26% of baseline mean score, respectively).

For the 2-min walk test, the three valid anchor-based MICs are 12.5 m (17% of baseline mean score). To the best of our knowledge, no MIC for the 2-minute walk test has been reported for older hospital

patients. However, Unnanuntana et al. [6] reported a very similar MIC of 12.7 m for the 2-minute walk test in 162 older patients recovering from total knee arthroplasty.

For the TUG, no valid anchor-based MICs could be established, and values ranged from 1.6 to 8.3 seconds. No MIC values for older hospital patients have been reported.

Anchor-based MIC values for the Barthel Index mobility subscale range from 2.5 to 7.5 (Figure 5), with 5/7 (71%) MIC values ≤ 3.5 points. Thus, a MIC of 3.5 points may be adequate (9% of the maximum scale range). However, two FAC-related anchor-based values were significantly larger (7.5 points), indicating a potentially higher and more robust MIC value for this instrument.

For the FAC, our results converged on a MIC of 0.5 points (10% of the 5-point scale range), indicating that a ≥ 1-point change in FAC is meaningful, since it can report whole numbers only. We are not aware of any FAC MIC estimations in any clinical population.

References Additional file 3

1. de Morton NA, Berlowitz DJ, Keating JL. A systematic review of mobility instruments and their measurement properties for older acute medical patients. Health Qual Life Outcomes. 2008;6:44.
2. Soares Menezes KVR, Auger C, Souza Menezes WR de, Guerra RO. Instruments to evaluate mobility capacity of older adults during hospitalization: A systematic review. Arch Gerontol Geriatr. 2017;72:67–79.
3. Perera S, Mody SH, Woodman RC, Studenski SA. Meaningful change and responsiveness in common physical performance measures in older adults. J Am Geriatr Soc. 2006;54:743–9.
4. Bohannon RW, Glenney SS. Minimal clinically important difference for change in comfortable gait speed of adults with pathology: a systematic review. J Eval Clin Pract. 2014;20:295–300.
5. Meretta BM, Whitney SL, Marchetti GF, Sparto PJ, Muirhead RJ. The five times sit to stand test: Responsiveness to change and concurrent validity in adults undergoing vestibular rehabilitation. J Vestib Res. 2006;16:233–43.
6. Unnanuntana A, Ruangsomboon P, Keesukpunt W. Validity and Responsiveness of the Two-Minute Walk Test for Measuring Functional Recovery After Total Knee Arthroplasty. J Arthroplasty. 2018;33:1737–44.
